# Imaginal disc growth factor maintains cuticle structure and controls melanization in the spot pattern formation of *Bombyx mori*

**DOI:** 10.1371/journal.pgen.1008980

**Published:** 2020-09-28

**Authors:** Yun Gao, Yun-Cai Liu, Shun-Ze Jia, Yan-Ting Liang, Yu Tang, Yu-Song Xu, Hideki Kawasaki, Hua-Bing Wang

**Affiliations:** 1 College of Animal Sciences, Zhejiang University, Hangzhou, China; 2 Faculty of Agriculture, Takasaki University of Health and Welfare, Gunma, Japan; The University of North Carolina at Chapel Hill, UNITED STATES

## Abstract

The complex stripes and patterns of insects play key roles in behavior and ecology. However, the fine-scale regulation mechanisms underlying pigment formation and morphological divergence remain largely unelucidated. Here we demonstrated that imaginal disc growth factor (IDGF) maintains cuticle structure and controls melanization in spot pattern formation of *Bombyx mori*. Moreover, our knockout experiments showed that IDGF is suggested to impact the expression levels of the ecdysone inducible transcription factor E75A and pleiotropic factors apt-like and Toll8/spz3, to further control the melanin metabolism. Furthermore, the untargeted metabolomics analyses revealed that BmIDGF significantly affected critical metabolites involved in phenylalanine, beta-alanine, purine, and tyrosine metabolism pathways. Our findings highlighted not only the universal function of IDGF to the maintenance of normal cuticle structure but also an underexplored space in the gene function affecting melanin formation. Therefore, this study furthers our understanding of insect pigment metabolism and melanin pattern polymorphisms.

## Author summary

The diverse stripe patterns of animals are usually used for warning or camouflage. However, the actual mechanisms underlying diverse stripe pattern formation remains largely unknown. This study provides direct evidence that imaginal disc growth factor (IDGF) maintains cuticle structure and controls melanization in the spot pattern formation. Our exhaustive knockout experiments reveal that BmIDGF is involved in the melanin pigmentation of *Bombyx mori*. We demonstrate that IDGF impacts the expression levels of the 20E-inducible transcription factor *E75A* and pleiotropic factors *apt-like* and *Toll8/spz3*, to further affect the melanin metabolism. Furthermore, the metabolome of *BmIDGF* gene deletion connects metabolism to gene function. Thus, this study shed light on not only the unique function of IDGF but also the molecular mechanism of spot pattern formation.

## Introduction

Animal spot and stripe patterns, which are typically observed pigmentation patterns, work as aposematic or camouflage coloration to help animals avoid predators. The convergent and divergent evolution of various stripe and spot phenotypes contributes to tracking environmental cues or escaping from unfavorable habitats, which provide a means for the study of adaptive morphological evolution. In insects, the pigmentation patterns of butterflies and moths play essential roles in processes such as mate preference, geographical adaptation, and mimicry [1–3]. It is worth noting that the color patterns of larval and adult bodies differ significantly not only between closely related species but also between different strains or life stages of the same species. Recently, research conducted on different species or spontaneous mutants within species has provided powerful evidence for the genetic and evolutionary regulation of the color patterns [2–9]. In those reports, various genetic factors that contribute to pigmentary phenotypic differences in larval and adult bodies have been identified, including numerous pleiotropic factors, the sex-linked gene *tan*, the HOX genes *abd-A* and *Abd-B*, and *Spatzle3* [10–12]. Although the formation mechanisms underlying the formation of stripe patterns of a variety of organisms have been studied, including zebrafish, zebra, rodents, and insects [13–16], the mechanism underlying the regulation of the metabolites for producing multiple stripe and spot patterns is largely unknown.

Lepidoptera is one of the most widely distributed animals on the planet and a major pest in agricultural production. A wide variety of markings and stripe patterns are found in Lepidoptera, the biological roles of which are more obvious and significant than those of other species. It is important to investigate the formation and regulatory mechanism of lepidopteran stripe and spot formation. The pigmentation synthesis for an emerging lepidopteran larva occurs mainly during the moulting period, which is controlled by 20-hydroxyecdysone (20E) [16,17]. Most lepidopteran body colors are comprised of various pigments, whereas melanin has the most common color patterns. Melanin is rooted in catecholamine precursors including 3,4-dihydroxy-L -phenylalanine (Dopa) and dopamine, which are produced by tyrosine. Among Lepidoptera, *B*. *mori* is an excellent model organism to investigate the molecular and genetic mechanism of color patterns because of its precise genome information, a high-density link-age map, and many available pigment deposition mutants in the egg, eye, wing, and body [18–21]. The melanin synthesis genes *Bmyellow*, *Bmebony* and *BmTH* were responsible for the silkworm color mutants, *chocolate* (*ch*), *sooty* (*so*) and *chocolate* (*sch*) loci, respectively [22]. The Toll ligand Spätzle3 and transcription factor Apontic-like (apt-like) contributed to the various stripe patterns primarily through triggering the gene expression profiles of *yellow* and *ebony* in *B*. *mori* and *Papilio xuthus* [9,11]. Although previous studies have identified the 20E signal and pleiotropic factors as the primary stripe-determining factors, the genetic scenario for diverse melanin pattern processes has remained unclear.

Lepidopteran larval color patterns principally rely on the nature and distribution of pigments in the epicuticle and epidermal cells. Compared with adult wings and bodies, larval pigmentation periodically accumulates, accompanied by the new cuticle that is replaced by the old cuticles at each ecdysis. Importantly, the cuticle of an insect is a multifunctional device and plays a vital role not only in maintaining the internal environment and supporting body shape, but also in protecting the organism against adverse environment conditions such as dehydration and the invasion of toxins, pathogens, and pesticides [23]. According to the biochemical and physiological composition of each functionally and morphologically distinct layer, insect cuticles are segmented into three layers: the outer waterproof envelope, the chitin-free and lipid/protein-rich epicuticle, and the inner procuticle containing a two-layer glycoprotein compound, exocuticle, and endocuticle [24–26]. However, the molecular mechanisms that control the assembly and replacement of the cuticle remain largely undetermined. Previous studies reported that imaginal disc growth factors that belong to the rarely known glyco-18-domain hydrolase family worked as a structural protein to maintain the extracellular matrix scaffold against chitinolytic degradation to stabilize the natural cuticle in *Drosophila* [27]. *Drosophila* has six *IDGF* genes that are involved in detoxification, innate immunity, and energy balance as well as in regulating temperature adaptation and development [27–29]. Intriguingly, a recent study of lepidopteran genome data revealed that lepidopteran insects have only one IDGF gene [30]. Some *IDGF* orthologues genes in Lepidoptera have been suggested to be involved in chitin matrix degradation during molting period [31–33]. However, the participation of IDGF in detailed physiological processes has not yet been elucidated.

To further investigate IDGF biological functions in Lepidoptera, we generated three *BmIDGF* mutants using the CRISPR/Cas9 system. We report insights from both transcriptional and metabolic investigations into the underlying function of BmIDGF for cuticularization and melanin synthesis. The homozygous larvae mutants exhibited the noticeable phenotype of disappearance of star spots, declining pigmented crescents, and deformed cuticle structures, which revealed that IDGF is involved in melanin synthesis and is indispensable for the formation of normal cuticles. Our results indicated that the interruption of *BmIDGF* caused the aberrant expression levels of 20E-inducible transcription factors E75A, apt-like and spz3, which specifically targeted downstream melanin synthesis pathway genes. We then exploit functional metabolomics to connect metabolism to gene function of *BmIDGF*. The untargeted metabolomics analysis found that the disruption of *BmIDGF* caused severe metabolic defects, which lead to a drop in accumulating melanin of the *BmIDGF* mutant. Our results describe, for the first time to the best of our knowledge, the pigmented phenotype of a structural protein that has long been known, in principle, to function in cuticle formation. The findings show that BmIDGF plays a critical role in spot-marking coloration and cuticular remolding, thus promoting our understanding of insect melanin pigmentation and morphological divergence.

## Results

### Phylogenetic analysis of IDGF

**Fig 1:**
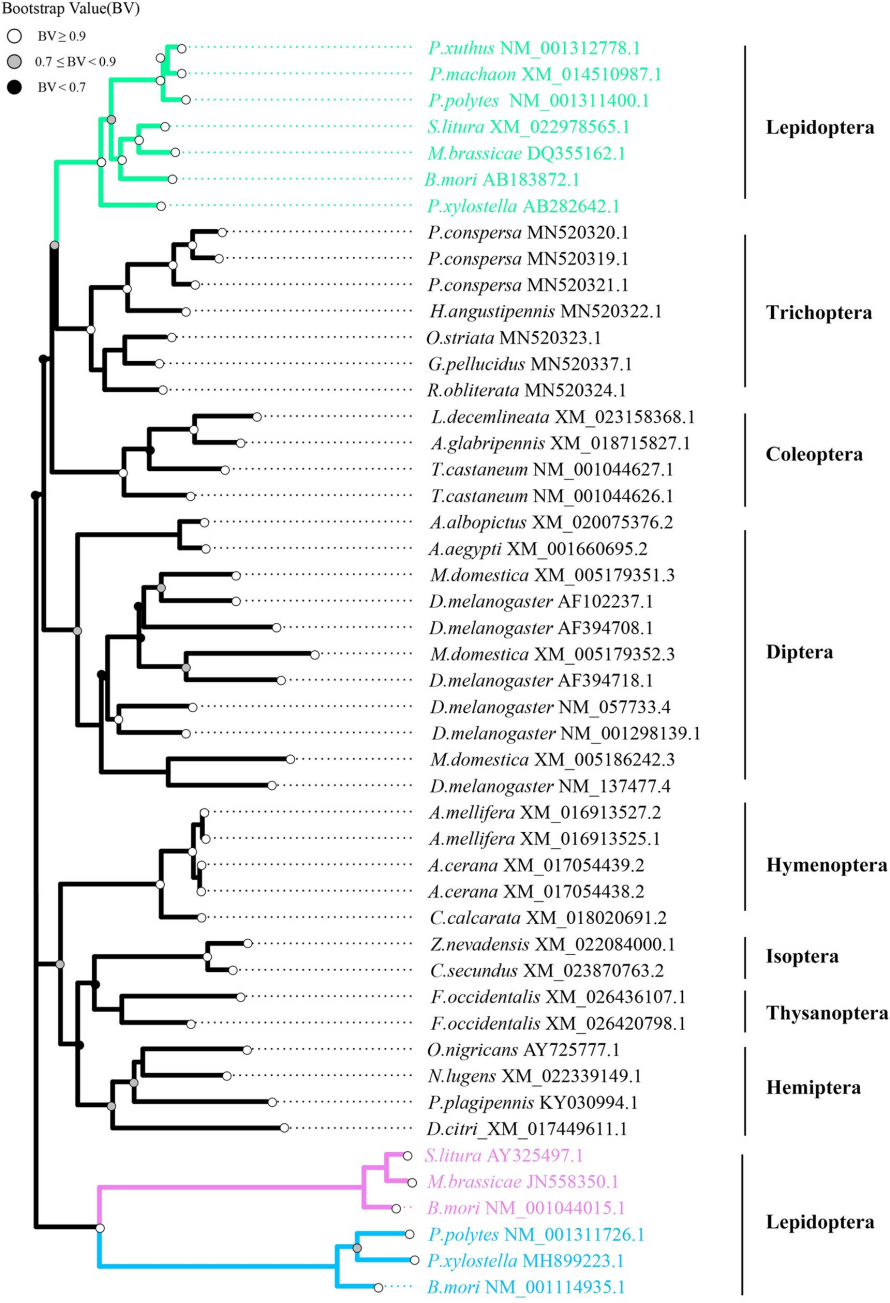
Phylogenetic analysis of *IDGF*. Unrooted maximum likelihood tree for IDGF and chitinase-like protein EN03 as well as chitinase was based on their codon sequences. The green branches represent lepidopteran *IDGF*s. The violet and blue indicate lepidopteran *chitinase-h* and *chitinase* genes, respectively. Branch lengths are scaled to the number of substitutions per site. Names and accessions used in the tree are listed in S1 Table.

To determine the evolutionary relationships of IDGF and chitinase-like protein in insect, a phylogenetic study was performed using the Maximum Likelihood (ML) method based on the full-length nucleotide sequence for IDGF and chitinase-like protein from several insect species. Phylogenetic analysis of a total of 48 IDGF and chitinase-like proteins resulted in eight distinct clades and well segregated from each other. Phylogenetic analysis indicated that there is a conserved monophyly group for *IDGF* among the lepidopteran species. The result suggested that *IDGF*s of Lepidoptera play conserved roles in developmental processes (**Fig 1**). In addition, the phylogenetic tree indicated that lepidopteran *IDGF*s were closely related to trichopteran *IDGF*s. Noteworthy, dipteran insects have evolved more than one IDGF, reflective of rapid evolution of this protein in Diptera. *Drosophila* has evolved six IDGFs, which serve various functions including organize the extracellular matrix formation of cuticles, involve in detoxification, innate immunity as well as energy balance and control temperature adaptation and development.

We conducted further multiple alignments among IDGF and chitinase-like proteins amino acid sequence of representative insect species to compare the functional domain. We found that the glyco-18 domain (motif1-12) was conserved among distantly species (Lepidoptera, Coleoptera, Diptera, Hemiptera, Isoptera, Hymenoptera), which suggests that all IDGF may evolve from the same ancestral sequence (**S1 Fig**). It is known that IDGFs belonging to the group V chitinase abrogated chitinase catalytic activity, because a glutamine residue substituted glutamate in the active catalytic domain, which is considered a highly conserved region of chitinases [34]. Here, our alignments of a total of 34 IDGFs and chitinase-like protein amino acid sequences indicated that the replacement was extremely well-conserved among Lepidoptera, Hemiptera, Hymenoptera, and Diptera (**S2 Fig**).

### *BmIDGF* mutant silkworm shows pigmentation defects

**Fig 2:**
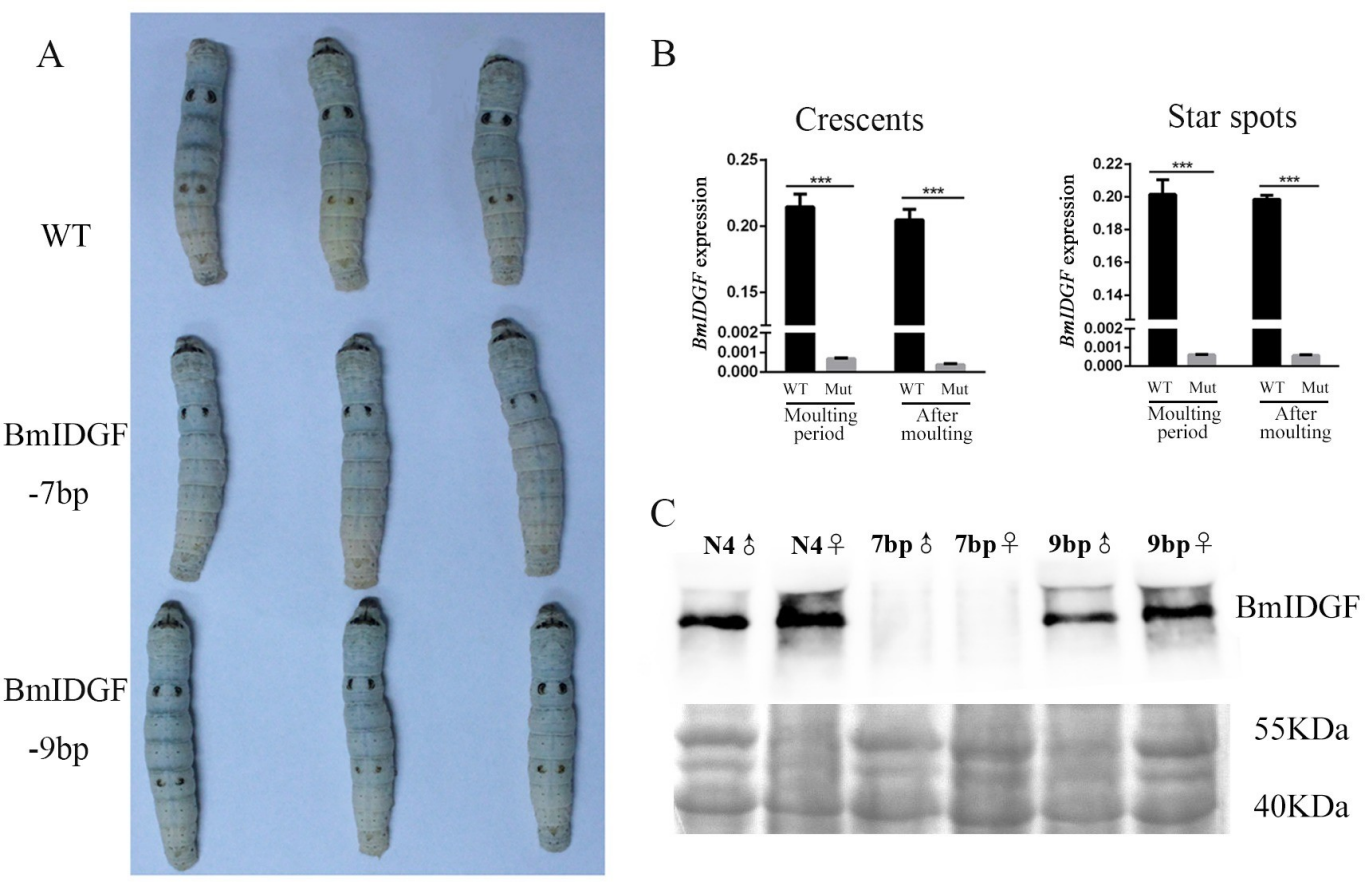
CRISPR/Cas9-mediated knockout of *BmIDGF*. (A) The disappearance of star spots and reduced pigmentation of crescents in the mutants when compared with the control silkworms after the fourth molting. (B) Significantly declining *BmIDGF* mRNA levels in the second abdominal segment (crescents) and the fifth (star spots) of mutants during the late fourth instar molting stage and after molting. The mRNA levels of *BmIDGF* were normalized by *Bmrp49* gene. *n* = 3. Error bars indicate mean value SEM. The asterisks indicate statistical significance (*p < 0.05, ** p < 0.01, *** p < 0.001, Student’s t test). WT, wild type; Mut, mutants. (C) Decrease in BmIDGF protein levels detected in the hemolymph in mutants of each sex. The IDGF antibodies detected the band for IDGF with the molecular sizes of approximately 48 kDa. Coomassie brilliant blue staining was used as a loading control.

To investigate physiological function of IDGF in silkworm, we genetically ablated *B*. *mori* IDGF using a Cas9/sgRNA-mediated mutagenesis system [35, 36]. Ultimately, three independent types of genomic deletions around the target sites were detected, which deleted 7bps, 8bps, and 9bps in the open reading frame (ORF) relative to the wild type and were defined as BmIDGF-7bp, BmIDGF-8bp, and BmIDGF-9bp, respectively (**S3 Fig**). The ORF of *BmIDGF* gene encodes a 433-aa peptide composed of the glyco-18 domain and the N-terminal signal-peptide for protein secretion. The BmIDGF-7bp line contains a 7-bp frameshift deletion at codon 230 that leads to the postponing of the translation start site but stops normally, which induces a truncated 255-aa protein with an extremely shortened glyco-18 domain and disappearance of N-terminal signal-peptide protein. The other two lines contain an 8- and 9-bp frameshift deletion at codons 223 and 228, resulting in amino acid chains with lengths of 397 and 430 aa, respectively. The homozygous mutants showed no deleterious phenotype compared with that of wild type individuals, and undergone normal larval-pupal metamorphosis as control larvae, which suggests that the disruption of *BmIDGF* did not interfere with the fertility and development of silkworms. Notably, homozygous BmIDGF-7bp and BmIDGF-8bp individuals significantly showed the phenotype of the disappearance of star spots and reduced pigmented crescents after the fourth molting. By contrast, the same phenomenon was not observed in the corresponding areas of the BmIDGF-9bp mutant (**Fig 2A**). In addition, the transcription level of *BmIDGF* was significantly lower in the second (crescents) and fifth (star spots areas) abdominal segments of the BmIDGF*-*7bp mutants than those of the wild-type control, demonstrating that this somatic mutagenesis system was effective (**Fig 2B** and **S4 Fig**). A complete disappearance in BmIDGF protein levels was also detected in the hemolymph dissected from the BmIDGF-7bp knockout animals, and the BmIDGF protein still could be found in the BmIDGF-9bp mutants (**Fig 2C**). These results suggest that the observed phenotype was caused by the decrease in the expression of BmIDGF in both the transcription and protein levels of the BmIDGF-7bp knockout insects. By contrast, the BmIDGF-9bp mutants seem to function as the control due to the lack of frameshift. Consequently, the line carrying the 7bp deletion was used for the subsequent experiments to investigate the function of *BmIDGF* and defined as *BmIDGF* mutant. The *BmIDGF* mutants were performed subsequent rescue experiments at the fourth instar as previously described [37]. The mutant larvae were injected with recombinant BmIDGF protein at the ratio of 200 ng protein/insect. As control group, the mutants were treated with an equal volume of PBS. The observed phenotype caused by BmIDGF protein injection showed no significant difference compared with the control group (**S5 Fig**).

### The BmIDGF mutant silkworm develops cuticularization defects

**Fig 3:**
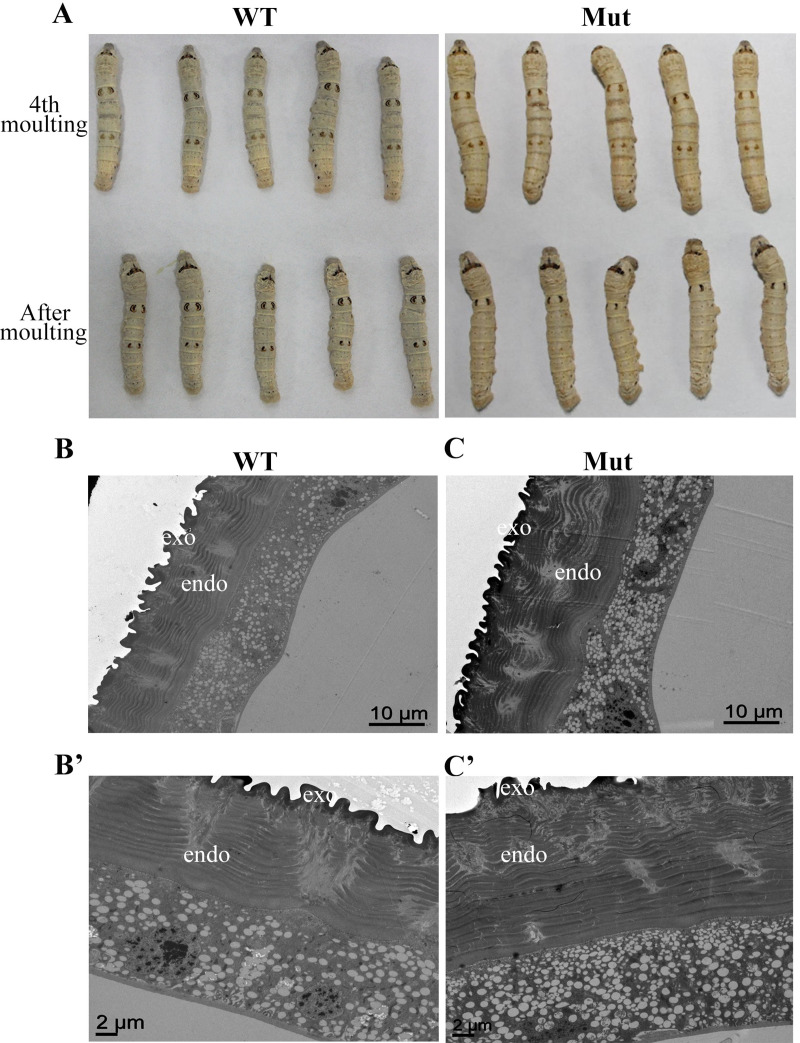
The comparison between the wild type and the mutants. (A) Larval marking phenotypes of the wild types and mutants, dorsal views of the wild strain (WT), and the *BmIDGF* mutant (Mut) of *B*. *mori* in the fourth molting (up) and after molting (down). Representative images show ultrastructure analysis of the larval body wall cuticle of the fifth abdominal segment from day three of the fifth instar (L5D3). The wild type epidermal extracellular matrix contains the lamellate chitinous procuticle, including the endocuticle (endo) and exocuticle (exo) (B and B′). The procuticle of BmIDGF knockout larvae contained more chitin-lamellae than those of the wild type (C and C′). Three biological replicates were performed for each disposition. Scale bars represent 10 and 2 μm in A and B, and A′ and B′, respectively. WT, wild type; Mut, mutants.

IDGF was found in the molting fluid, and it may have been conducive to the stability and protection of the growing procuticle [27, 33]. For gaining better understanding of lepidopteran IDGF in functioning cuticle barriers, we performed the Transmission election microscopy (TEM) using the larval epidermis of the fifth abdominal segment of 5th instar larvae 72 ± 3 h after ecdysis. The wild insect cuticle usually consists of multiple layers, including the envelope, outermost epicuticle (outer), exocuticle (medial), and endocuticle (inner). The exocuticle that is part of the new cuticle deposited during a brief period before ecdysis, and the endocuticle then becomes thicker with each chitinous layer deposition [25, 26]. The *BmIDGF* mutant larvae showed defects in cuticle ultrastructure with thicker endocuticles compared with the wild-type control. This abnormal cuticle had more endocuticular lamellae that were rich in protein/chitin than the wild-type group, suggesting that the degradation of old chitin-lamellae was disturbed during molting period (**Fig 3** and **S6 Fig**). Furthermore, more uric acid particles were found in the epidermal cells of the mutants relative to wild-type control, which coincided with the phenomenon that epidermal cells under the white stripes contained many uric acid granules in several lepidopteran larvae. These results suggest that IDGF is indispensable for the normal formation of the endocuticle.

### BmIDGF affects the expression of melanin synthesis pathway genes

**Fig 4:**
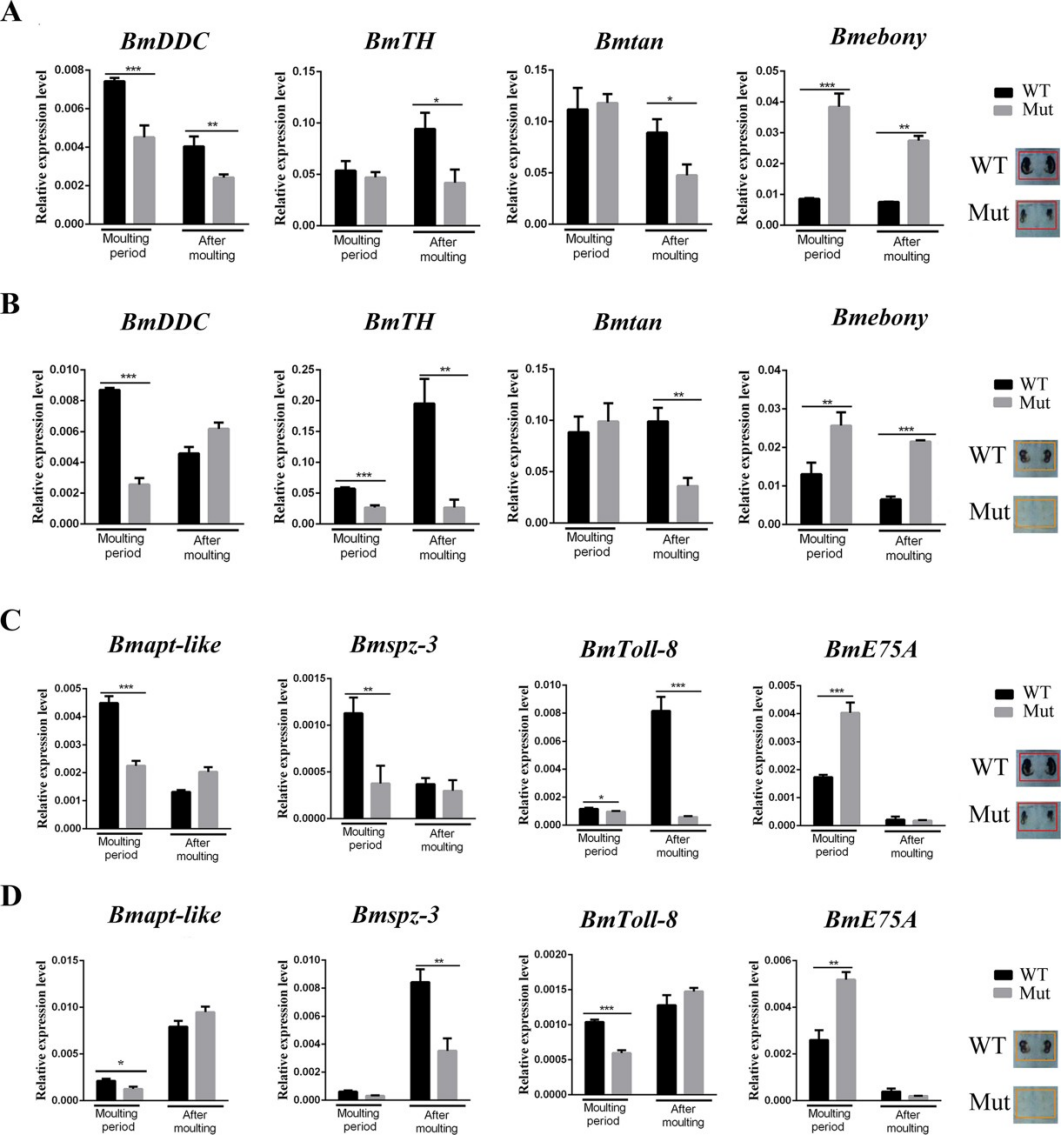
Expression profiles of melanin synthesis-related pathway genes of the larval epidermis by qRT-PCR. Relative expression levels of melanin synthesis-related pathway genes between the wild type and the mutants were determined by quantitative RT–PCR analysis. Given are the expression patterns of melanin synthesis pathway genes in the larval epidermis from the late fourth instar molting and just after the fourth instar molting in the crescents (A) and star spots (B). The temporal expression patterns of four genes upstream of the melanin synthesis pathway in the crescents (C) and the star spots (D) from the *BmIDGF* mutant and the wild strain. WT, wild type; Mut, mutants. *n* = 3. Error bars indicated mean value SEM*p < 0.05, ** p < 0.01, *** p < 0.001 (Student’s t test).

Larval color patterns are largely determined by the nature and distribution of pigments in procuticle layer and exocuticle. The wild type strain includes three types of spots: star spots on the fifth abdominal segment, crescents on the second abdominal segment, and eye spots on the second thoracic segment [9]. After the fourth molting, the visible changes of declining pigmented crescents and disappearance of star spots were found in the *BmIDGF* mutant strain (**Fig 3A**). To gain further insight into the cause of phenotypic differences associated with the *BmIDGF* mutants and the wild type, we analyzed and compared the spatiotemporal expression profiles of melanin synthesis pathway genes, including two secreted protein genes *yellow* and *laccase2*, as well as the epidermal melanin synthesis enzyme genes *ebony*, *tan*, *TH*, and *DDC*. During the fourth instar molting and the fifth instar, we prepared cDNA from the larval epidermis of the second and fifth abdominal segments, for which pigmentation occurs in the wild type, but whose corresponding areas decreased and disappeared, respectively, in the *BmIDGF* mutants. During the fourth molting, we found that the *BmDDC* mRNA expression level of crescents was lower in the *BmIDGF* mutant strain than in the corresponding areas of the wild-type control. The mRNA expression level of *Bmebony* was higher in the *BmIDGF* mutants than in the wild type (**Fig 4A**). Similar expression patterns of *BmDDC* and *Bmebony* were also detected in the star spots area (**Fig 4A** and **4B** and **S7A** and **S7B Fig**). It is known that TH catalyzes tyrosine to Dopa, which is the main precursor for insect melanin. Unexpectedly, the expression of the vital gene *BmTH* that encodes TH was equivalent in the crescents in both strains during molting, but a significant reduction of this gene was observed in the star spots region of the mutants (**Fig 4A** and **4B** and **S7A** and **S7B Fig**). These results show that the interruption of *BmIDGF* influenced the melanin synthesis pathway, primarily *BmTH*, *BmDDC*, and *Bmebony* genes, which impaired the region-specific pigmentation of crescents and star spots in the mutants.

### BmIDGF is involved in the melanin pigmentation

Previous reports revealed that the 20E-inducible transcription factor and pleiotropic factors the pleiotropic regulatory factor Toll ligand Spätzle3, and the transcription factor apt-like can regulate melanin synthesis pathway genes [9,11,17]. To determine whether the defects in the melanin synthesis pathway could be attributed to disruption of the upstream regulatory network, we performed temporal and spatial expression analysis of the above genes (**Fig 4C** and **4D** and **S7C** and **S7D Fig**). The boosted expression of *BmE75A* was observed during molting period when the melanin pigmentation occurred. Moreover, the mRNA expression levels of *Bmspz3*, *BmToll8*, and *Bmapt-like* were significantly lower in the crescent marking regions of the *BmIDGF* mutant strain compared with those in the corresponding regions of the wild-type control (**Fig 4C** and **S7C Fig**). Similar expression patterns of BmToll8 were observed in the star spots of the *BmIDGF* mutants (**Fig 4D** and **S7D Fig**). The above results showed that *BmIDGF* prominently influenced *BmE75A*, *Bmapt-like*, and *Bmspz3/Toll8* expression levels, which further affected the melanin synthesis pathway genes *BmTH*, *BmDDC*, and *Bmebony* to restrain region-specific pigmentation. Altogether, these findings suggest that BmIDGF affects the gene expression of the melanin synthesis pathway during the pigmentation processes.

### The absence of BmIDGF provokes significantly metabolic change

Previously, we purified IDGF with apolipophorin-I/II from silkworm hemolymph, which suggests that the BmIDGF protein is transported in the hemolymph and may be involved in the transportation of molecules of crucial physiological processes [32]. To better understand how the interruption of BmIDGF influenced metabolic mechanisms, we generated dynamic metabolome profiles of hemolymph collected at three time points (fourth instar, fourth instar molting, and fifth instar). The polar metabolites were extracted from hemolymphs at each time point and analyzed by non-targeted flow-injection metabolomics [38].

The untargeted liquid chromatography-mass spectrometry (LC-MS) analyses yielded a total of 6894 metabolites. 5552 metabolites satisfied with the relative standard deviation (RSD)≤30% were used for further statistical analysis. The untargeted metabolomics data were initially employed by principal component analysis (PCA) to acquire a different global view of the data and showed that the first two principal components (PCs) explained 46.83% of the total variation. It is remarkable that the differential metabolites of the fourth instar molting were clearly distinguished from those of the remaining two time points (**S8 Fig**). Moreover, the two PCs identified several individual metabolites whose expression variations make a major contribution to each PC. Importantly, the partial least squares discrimination analysis (PLS-DA) between the wild type and the *BmIDGF* mutant was carried out for the LC-MS data. The variable importance in projection (VIP) value of the two PCs for the PLS-DA model and a combination of fold change and q-value revealed a total of 581 significantly differential accumulated metabolites (fold change ≥ 1.2 or ≤ 0.8333, q-value < 0.05, VIP ≥ 1) at three time points as previously described [38]. Unsupervised hierarchical clustering of the 581 metabolite abundance changes, using the Euclidean distance as a similarity metric after standardization, suggests overall gradual changes in metabolite expression over time (**S9 Fig**). The global expression pattern yielded three different temporal clusters corresponding to fourth instar, a melanin synthesis process fourth instar molting, and fifth instar. Specifically, the distinct metabolites identified from the fourth molting are significantly different from those from the other two stages, which is consistent with the PCA analysis.

To gain comprehensive understanding of metabolic alterations at different time points between the wild type and the mutants, we next assigned 581 differentially accumulated metabolites to pathways based on the Kyoto Encyclopedia of Genes and Genomes (KEGG) database using the Molecule Annotation System. The KEGG analysis comparing the wild type and mutant samples revealed significant enrichment for several pathways, including glycometabolism, amino acid metabolism, metabolic pathways, ascorbate and aldarate metabolism, phenylalanine metabolism, purine metabolism, and tyrosine and pyrimidine metabolism at three time points (**S10 Fig**). Collectively, these data provide a general resource on the dynamics occurring in the metabolome of the mutants.

### The metabolome of *BmIDGF* gene deletion connects metabolism to gene function

The black markings of insect cuticle were composed of polymeric melanin pigments. The melanin synthesis process could be divided into two aspects, the generation of tyrosine in the phenylalanine metabolism pathway and the conversion of tyrosine into melanin in the tyrosine metabolism pathway [11]. Physiological phenotypes analysis showed that the break of BmIDGF resulted in a drop in the melanin pigmentation—while all other traits remained steady, we assessed the consequences of decreased melanin on metabolism.

**Fig 5:**
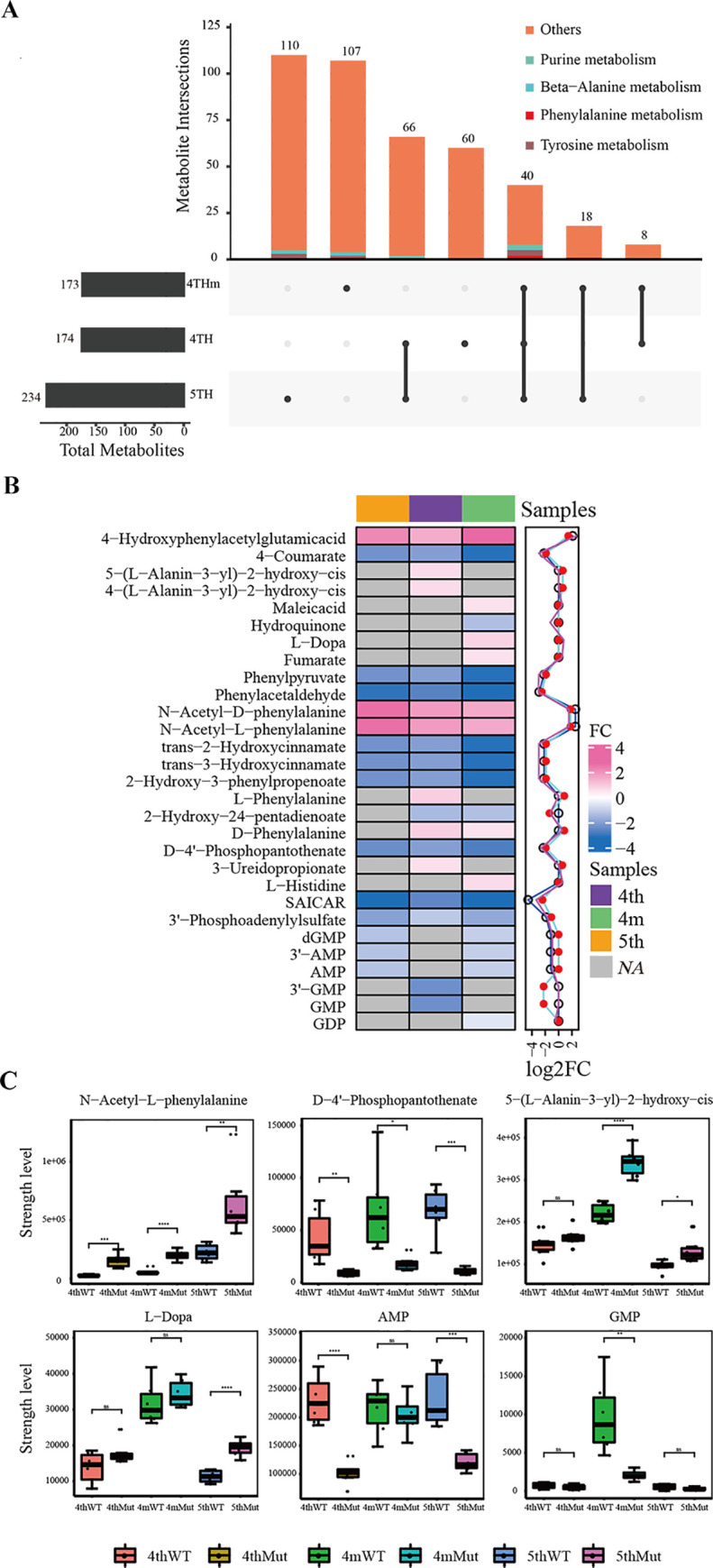
Metabolomic changes following interruption of BmIDGF. Relative abundances of differential metabolites of phenylalanine, beta-alanine, purine, and tyrosine metabolism pathways were compared between the wild type and the mutants. (A) Histogram showing the distribution of different metabolites at three different time points. (B) The heat map shows the different abundance of metabolites belonging to the above pathways between the wild type and the mutants at three stages. Shades from blue to pink represent the increasing metabolite levels. Gray shades are metabolites with no significance. (C) Abundance plot of crucial metabolites in the hemolymph of the wild type strain and the BmIDGF mutant strain. Data were derived from metabolomics assay data. Box plots are whiskers according to the strength level of crucial metabolites. Different color of boxes represent different stages of mutants and wild type. The color codes for the boxes are labeled on the picture. The orange and yellow, green and wathet, mazarine and rouge color denote samples of wild type and mutants at fourth instar, fourth instar moulting and fifth instar, respectively. WT, wild type; Mut, mutants. *n* = 6. Error bars indicated mean value SEM. *p < 0.05, ** p < 0.01, *** p < 0.001 (Student’s t test).

To obtain a systematic view on the synthetic and metabolic destiny of melanin, we analyzed the differential metabolites identified from the LC-MC data. The histogram shows a total of 60, 107, and 110 metabolites identified from the fourth instar, fourth instar molting, and fifth instar samples. Notably, a total of 40 metabolites, which belong to the abovementioned metabolism pathways, were commonly observed across all sampling times. Several distinct metabolites (5-(L-alanin-3-yl)-2-hydroxy-cis, 4-(L-alanin-3-yl)-2-hydroxy-cis, and GMP) that belonged to tyrosine and purine metabolism were only found in the fourth molting when the melanin was synthesized and deposited in the cuticle (**Fig 5A**). Out of 581 distinct metabolites, 18 increased significantly, and 13 were less abundant in melanin synthesis-related metabolic pathways of the mutants, of which two, L-Dopa and 5-(L-alanin-3-yl)-2-hydroxy-cis, belonged to the same metabolic pathway, the tyrosine metabolism pathway (**S8B–S8D Fig**). Thus, we focused on the altered metabolites of the surrounding metabolic network, including the phenylalanine, tyrosine, beta-alanine, and purine metabolism pathways. The heat map showed a large number of metabolites that exhibited differences in abundance and that were associated with the above pathways at three different time points (**Fig 5B**). We performed a phenylalanine metabolism pathway analysis and found that N-acetyl-L-phenylalanine, the downstream product of phenylalanine, increased 2.8- to 5.7-fold at all-time points in the *BmIDGF* mutants as compared with the wild-type control (**Fig 5C**). During molting, L-Dopa was rapidly converted into melanin in the primary tyrosine metabolism process. 5-(L-alanin-3-yl)-2-hydroxy-cis and 4-(L-alanin-3-yl)-2-hydroxy-cis, which are downstream of L-Dopa with a role in producing O Stizolobate and O Stizolobinate, were more abundant in the *BmIDGF* mutants than those in the wild type (**Fig 5C**). These data suggest that L-Dopa was heavily converted into O Stizolobate and O Stizolobinate instead of eumelanin, which was the fatally important precursor of insect melanin. Beta-alanine played a crucial role in the sclerotization pigmentation of the new cuticle. In particular, D-4′-phosphopantothenate, a downstream product of beta-alanine, showed decreased accumulation in the *BmIDGF* mutants at three time points, suggesting that less beta-alanine was consumed (**Fig 5C**). The redundant beta-alanine appeared to bind with L-Dopa to yield yellow N-β-alanyl dopamine (NBAD), which lessened the amount of L-Dopa used for melanin synthesis. We were intrigued by the trends in the significant increase of L-Dopa in the *BmIDGF* mutants at the fifth instar when the pigmentation had been completed, which implied that less L-Dopa was used for melanin synthesis during molting. Importantly, the KEGG pathway analysis revealed that several differential metabolites were significantly enriched in the purine metabolism pathway (**S10 Fig**).

Uric acid was the final product of purine metabolism and was stored in the epidermis by storage excretion [39]. It was of great significance to find that several metabolites, including AMP, GMP, 3′-AMP, and 3′-GMP, which are required for the generation of uric acid, were sharply decreased in the *BmIDGF* mutants at three time points (**Fig 5C**). The results suggest that the metabolites of the purine metabolism pathway were largely consumed to generate uric acid in the mutants, which was consistent with the irregular integument structure with abundant uric acid granules in the *BmIDGF* mutants relative to the wild type. The absence of *BmIDGF* induced metabolic changes toward a decrease in melanin accumulation, dependent on the tyrosine, phenylalanine, beta-alanine, and purine metabolism pathways. Taken together, we concluded that *BmIDGF* is participating in gene-metabolism interactions that enable access to an underexplored space in *BmIDGF* gene function.

## Discussion

In this study, we used an efficient CRISPR/Cas9 system to knockout *BmIDGF* to expound the function of lepidopteran IDGF. After the fourth molting, the disappearance of star spots and weak pigmentation of crescents were significantly observed in the homozygous *BmIDGF* mutant larvae. The cuticular ultrastructure analysis showed that more chitinous layers and uric acid particles were observed in the *BmIDGF* mutants than those of the wild-type control. We then confirmed that the disruption of *BmIDGF* affected the genetic circuit of *BmE75A*, *Bmapt-like*, and *Bmspz3/Toll8*, which contributed to the phenotypic change of silkworm spot patterns by specifically targeting gene expression in melanin synthesis genes, including *BmDDC*, *Bmebony*, and *BmTH*. Furthermore, the untargeted metabolomics data demonstrated that the interruption of *BmIDGF* exerted pleiotropic effects on several metabolic pathways, including the tyrosine, phenylalanine, beta-alanine, and purine metabolism pathways, which skewed the metabolism in the *BmIDGF* mutants toward a decrease in melanin accumulation. We conclude that lepidopteran IDGF is indispensable not only for chitinous layer degradation of the cuticle but also for larval spot color patterns through coordinated regulation of melanin metabolism.

The glycoside hydrolase 18 (GH 18) family contains a large number of members, including chitinases with catalytic activity and IDGFs without catalytic activity, which play diverse roles during insect cuticle development and molting [26–29, 31,32]. Phylogenetic analysis indicates that all lepidopteran IDGFs comprise a monophyletic group. Notably, the monophyletic IDGF homologs of Lepidoptera possess highly conserved structures not only in the Gly-18 domain but also in the N-terminal signal-peptide (**S1** and **S2 Figs**). Our results suggest that the function of IDGF has been shown to be conserved, at least among Lepidoptera. Here, we found that *BmIDGF*-knockout larvae showed a malformed cuticle structure, similar to the defective procuticle structure of *Drosophila* IDGF RNAi mutants [27]. More wrinkles were observed in the whole body of the *BmIDGF* mutants after the molting, suggesting cuticle defect occurred in whole body. Likewise, *B*. *dorsalis* IDGF4 also plays an important role in cuticle formation and the organization of proteins in the chitin matrix [29]. It is worth noting that four chitinases, BmIDGF, BmChtI, BmChtII, and BmChi-h, were found in the molting fluid of *B*. *mori* [33]. Although IDGF abrogates chitinase catalytic activity, its disruption may influence the degradation of the old exoskeleton and formation of a new cuticle. We have demonstrated a simple genetic basis for structural coloration and provided the first phenotypic demonstration of the contribution of IDGF to insect pigmentation. In addition, our previous study indicated that BmIDGF was observed in several tissues, such as the epidermis, brain, testis, and ovary [32]. These findings imply that lepidopteran IDGF has crucial functions for various morphological incidents other than larval cuticle formation.

The most important finding of this study was that the absence of *BmIDGF* resulted in the loss of pigmentation in the *BmIDGF* mutants with the reduced pigmentation of crescents and disappearance of star spots, suggesting that *BmIDGF* likely interacts with the melanin synthesis pathway and contributes to the phenotypic diversity of larvae. In several *Drosophila* species, changes in the spatial expression patterns of melanin synthesis-related genes, including *yellow*, *tan*, and *ebony*, result in species-specific color pattern diversity [2, 40, 41]. The alteration of color patterns of Lepidoptera was considered a complex developmental course precisely modified by multiple hormonal behaviors in a continuous order [42]. In this study, our experiments reveal that the especial phenotype of reduced pigmentation of crescents and disappearance of star spots is strongly correlated with the abnormal expression level of the melanin synthesis genes *BmDDC*, *BmTH*, and *Bmebony* (**Figs 3A**, **4A** and **4B**).

In addition, the *E75A* transcript was persistently highly expressed during molting period when the melanin deposited, suggesting that the continually high expression level of BmE75A may suppress downstream melanin synthesis pathway gene expression (**Fig 4C and 4D**). The pleiotropic regulatory factors, including *apt-like*, worked as upstream elements of caterpillar melanin synthesis pathways to regulate the melanin synthesis module in a stage-specific and region-specific manner [9,11]. Here, we found that the expression levels of *Bmspz3*, *BmToll8*, and *Bmapt-like* were significantly lower in the crescent marking regions of the *BmIDGF* mutants compared with the wild strain. We conjectured that Bmapt-like in the protease cascade of *BmIDGF* processing enabled the activated BmIDGF protein only in the future spots’ regions. These results indicate that these melanin synthesis genes act as a module that was coinfluenced by BmIDGF. Although the genes and gene networks involved in the regulation of *BmIDGF* remain unclarified, the elevated *BmE75A* and damped *Bmapt-like* and *Bmspz3/Toll8* are considered to account for the abnormal spot stripes of the mutants. Previous studies reveal that there are many melanin synthesis pathway genes and intermediate metabolites involved in melanin synthesis. We found that the *BmIDGF* mutants still showed some markings in other segments. We speculate that there is a certain compensation mechanism of melanin synthesis in the mutants, which lead to the different phenotypes of the spots. The mechanism needs further clarification.

**Fig 6:**
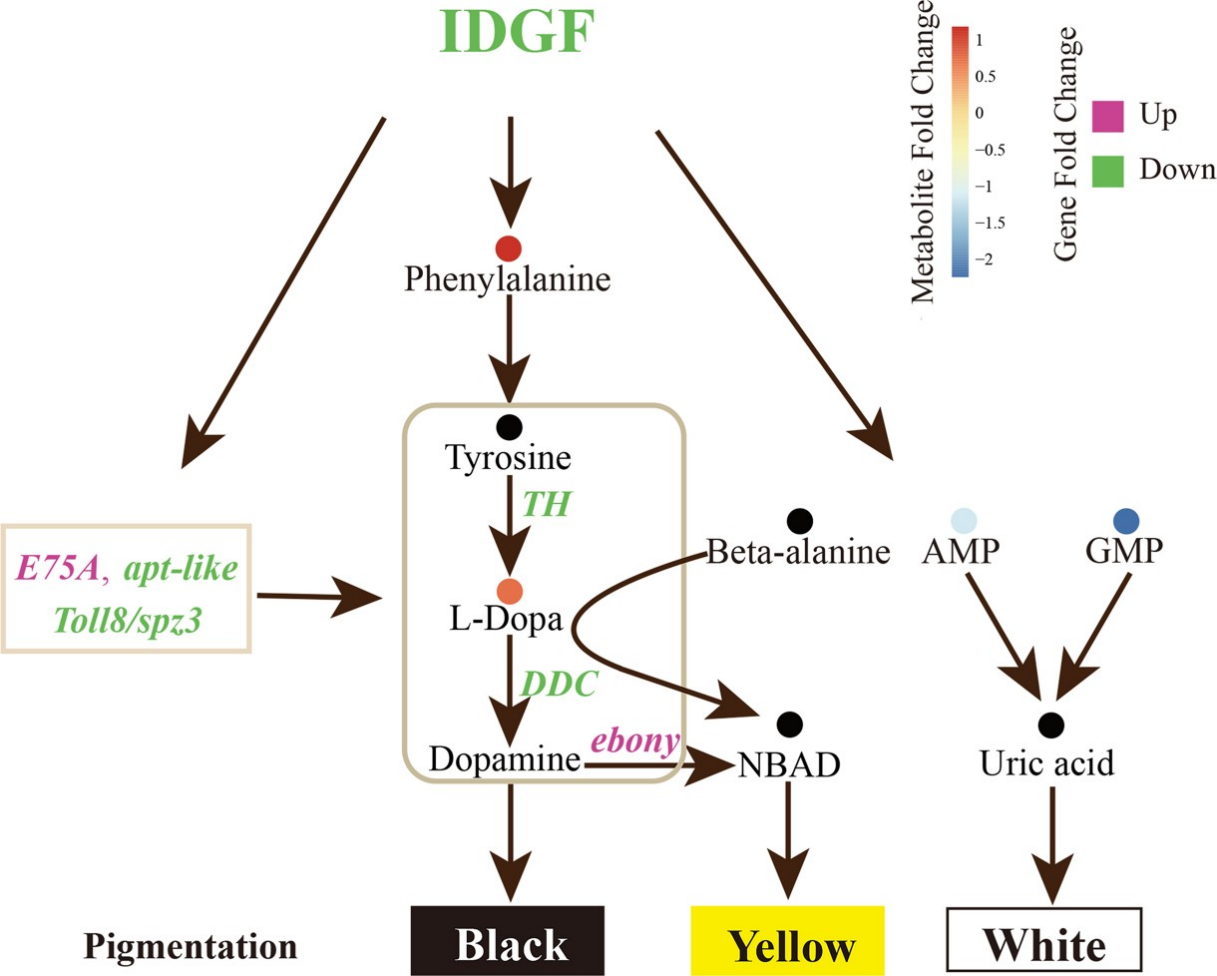
A model of the pathway involved in melanin formation and larval pigmentation. The italic rosy (increased) and green (decreased) letters represent the markedly alternated genes of melanin synthesis pathways in crescents and star spots areas during the moulting. The metabolites of hemolymp whose abundance changed significantly are in colored circles. Dots from blue to orange represent the increasing metabolite levels. Black dots are metabolites that were not detected by MS.

Our study provides an illustrative instance of how untargeted metabolomics analyses can result in biological insights with great practical significance. Suggestive evidence for a link between IDGF and the organismal metabolism was also provided by earlier findings. BmIDGF was purified together with apolipophorin-I/II from *B*. *mori* hemolymph and played a critical role in glucose metabolism and development in silkworms [32]. This enabled us to exploit the functional metabolomics to connect metabolism with gene function. On the basis of the non-targeted flow-injection metabolomics analysis of hemolymph at three time points, we observed that the interruption of BmIDGF largely caused metabolism disorder, and we inspected the changes in melanin metabolism more closely to assess the consequences of decreasing melanin accumulation. Although the mechanism by which IDGF induced broad metabolic changes remains elusive, a possible explanation for the switch toward decreasing melanin accumulation is that the absence of IDGF downregulates the phenylalanine and tyrosine metabolism pathways, which have been shown to fuel the melanin synthesis and consequently deposit melanin into the cuticle. The abnormal metabolism not only enhanced the consumption of L-Dopa but also generated a yellow cuticle [43]. On the basis of earlier studies and our results, we submit a possible model of the genic and metabolic network among IDGF and melanin biosynthesis in **Fig 6**. During the fourth molting, the decreased expression levels of *BmTH* and *BmDDC* in the *BmIDGF* mutants restrained the physiological process of the conversion of tyrosine into Dopa and subsequent conversion of Dopa into Dopamine. In addition, a large amount of the important melanin precursor Dopamine was selectively converted into NBAD by the elevated expression of *Bmebony*. On the other side, the redundant β-alanine bound to L-Dopa to yield NBAD. Indeed, the disturbance of BmIDGF showed a strong accumulation of L-Dopa after the fourth molting. Moreover, the increase of L-Dopa’s downstream metabolites, 5-(L-alanin-3-yl)-2-hydroxy-cis and 4-(L-alanin-3-yl)-2-hydroxy-cis, were observed during molting, which may be the consequence of the decreased expression level of BmDDC that converts L-Dopa into Dopamine. Furthermore, a fourfold decrease of D-4′-phosphopantothenate concentration, which is the downstream metabolite of beta-alanine, appears to induce redundant beta-alanine binding with L-Dopa to generate yellow NBAD. These data indicated that L-Dopa was less consumed, and large amounts of L-Dopa’s downstream metabolite, Dopamine, were used to produce NBAD, which caused the reduction or absence of spot pigmentation. These results suggest that BmIDGF is participating in gene-metabolism interactions and involved in the melanization process.

Alterations of the purine metabolism pathway were another striking effect of the absence of BmIDGF levels. Uric acid is a major waste product of nitrogen metabolism in terrestrial insects. The key metabolites AMP and GMP required for producing uric acid were dramatically decreased at three time points in the hemolymph of the mutants, suggesting that they were heavily consumed and promptly converted into uric acid. These results are consistent with the findings that the *BmIDGF* mutants had more uric acid particles in the deformed cuticle (**Fig 3B**). Notably, previous results revealed that uric acid granules in the epidermis are a crucial component of body coloration since they caused the larval integument to become opaque or white. Thus, this study uncovers the unique aspect of IDGF function in spot pattern pigmentation and establishes an amusing metabolic process for the melanin synthesis. Future investigations that exploit such important metabolic intermediates will further our understanding of the molecular mechanisms underlying the formation and regulation of melanin synthesis.

In conclusion, our results reveal that IDGF contributes to cuticle assembly and organization, and melanin formation in *B*. *mori* and perhaps other insects as well. In addition, the metabolome of *BmIDGF* gene deletion connects metabolism to gene function. Our dataset on the dynamics of the metabolome for melanin synthesis provides comprehensive understanding of the function of IDGF on metabolism. Therefore, this study will lead to greater understanding of insect pigment metabolism and morphological divergence.

## Materials and methods

A multivoltine and oligophagous silkworm strain, N4, were reared on mulberry leaves or an artificial diet at 26°C and relative humidity of 75% under long-day conditions (12 h light:12 h dark).

### Phylogenetic analysis

To illuminate the distribution and the evolutionary relationships of IDGFs and chitinase-like proteins in insect species, the phylogenetic analysis was performed using the protein sequences included in the Blastp hits (E-value<10^−4^) on the NCBI server (http://www.ncbi.nlm.nih.gov/BLAST/) as described previously [9, 44, 45]. The amino acid of BmIDGF were used as a query for Blastp search. The sequences used to create the diagram were listed in **S1 Table**. The phylogenetic tree was constructed using the maximum likelihood method through the MEGA7 software (GTR + I+G model). The confidence levels of various phylogenetic lineages were assessed by bootstrap analysis (1,000 replicates). Phylogenetic tree was visualized online using Interactive Tree of Life (iTOL) (http://itol.embl.de) and annotated by the R package “ggtree” [46]. Besides, the protein domain analysis was performed following the previously study [47].

### CRISPR/Cas9-mediated mutagenesis in *B*. *mori*

CRISPR/Cas9 system was used as an effective approach to knockout targeted gene of *B*. *mori* [35, 36, 48]. According to the 5′-GG-N_17/18_-NGG-3′ rule, the sgRNA target site was designed by manually searching genomic regions on the website. The potential off-target binding of sgRNA sequences were tested bioinformatically by CRISPRdirect (http://crispr.dbcls.jp/) through exhaustive searches against silkworm genomic sequences for selecting the best sequences. Cas9 mRNA and sgRNA stands for single guide RNA were synthesized and purified using T7 MEGAscript kit (Ambion) and mMessagemMachine T7 kit (Ambion), respectively. A mixture of Cas9 mRNA (300 ng/μl) and *BmIDGF* sgRNA (100 ng/μl) was injected into the embryos prepared within 6 h after oviposition using a micro-injector. The injected embryos were incubated at 25°C in a humidified chamber for 9–10 days till larval hatching, and the hatched larvae were reared normally with fresh mulberry at the circumstance of 12h:12h (light: dark), 26°C and relative humidity of 75%. For the sgRNA site, somatic mutagenesis was detected by PCR-based analysis using gene-specific primers which set on the upstream or downstream of each target and subsequent sequencing. Mutants were generated at a single site, indicating that successful mutagenesis was induced by the transgenic CRISPR/Cas9 system. To attain the mutants who are stable heritable homologous to assess precisely functional analyses, a series of crossing strategies and persistent PCR-based multigenerational selection were performed to ensure the germline transmission of the genotype.

### Western blotting

Epidermis was dissected and washed in pre-cool phosphate-buffered saline (PBS) as well as grinded in RP lysate (RIPA: PMS = 100:1), then incubated on ice for 30 min. Centrifuge the mixture with 15,000 rpm for 15 min at 4°C. The supernatant (extracted protein) was quantitative using BCA method. The hemolymph was collected and centrifuged with 4000 rpm for 5 min, then remove sectional supernatant and the rest (extracted protein) was quantitative using BCA Kit. 20 μg total protein was separated by SDS-PAGE and transferred into PVDF membranes, blocked in 5% nonfat milk for an hour. The primary antibody of BmIDGF was made as previously described [32]. Then the membranes were incubated with polyclonal rabbit anti-BmIDGF primary antibodyfor specifically detecting BmIDGF (1:1000 dilution) and washed with TBST (50 mM Tris, 150 mM NaCl and 0.1% Tween-20). The goat-anti-rabbit lgG antibody purchased from company (Thermo Fisher Scientific, USA). Membranes labeled with primary antibodies were incubated with goat-anti-rabbit lgG antibody at a 1:5,000 dilutions for one hour and washed with TBST for 15 min. At last, membranes were visualized using the Chemi Capture (CLINX-science instruments, Shanghai) and images obtained were processed using Photoshop S6. As for control, the same samples were also detected via Coomassie Brilliant Blue (CBB) staining. The western blotting and CBB images are representatives of more than 3 independent biological samples.

### Expression and purification of the recombinant BmIDGF protein and injection

The recombinant BmIDGF was produced using a Bac-to-Bac system (Invitrogen) as described previously [49]. The primers used to construct the vectors are listed in **S2 Table**. Briefly, the coding region of *BmIDGF* with a His-tag sequence at the C-terminus subcloned into the donor plasmid pFastBac1 (Invitrogen) and transfected into Sf 9 cells. Recombinant BmIDGF was purified by HisSpinTrap (GE Healthcare). The rescue experiment was conducted according to the previous research [37]. The homozygous mutant larvae were injected with BmIDGF protein at the ratio of 200 ng protein/insect or equal volume of PBS. Each treatment was conducted in 3 biologic replication.

### Quantitative reverse transcription-PCR (qRT-PCR)

The second (the crescents) and fifth (star spots) abdominal segment was dissected 4–22 h after head capsule slippage (HCS) at the fourth molting and after molting stages [9]. Total RNA was isolated using RNAiso Plus (Takara, Japan) and 1 μg of total RNA of each sample was reverse transcribed using PrimerScript RT Reagent Kit with gDNA Eraser (Takara, Japan) according to the manufacturer’s instructions. Real-time PCR was performed to analyze specific genes’ expression profile as described previously [49]. The specific primers used for Real-time PCR were designed by Primer 5 software and listed in **S2 Table**. Expression levels of each sample were demonstrated with more than three biological replicates. The gene for the ribosomal protein 49 (*rpl49*) was used as an internal control to normalize the loading of equal sample. Student's t-test was used for evaluating the statistical significance.

### LC-MS sample preparation

For steady-state hemolymph metabolite profiling, hemolymph (>100 μL) of mutants and wild type (6 repetitions of each type) were collected on ice at the time of fourth instar, fourth instar mouthing and fifth instar, 40 μl of each sample was taken into the corresponding 96-well plate. After adding 120 μl of pre-cooled methanol, the sample was mixed and shacked for 1 min and then placed under the circumstance of -20°C for 2 h or overnight. Next, the mixtures were centrifuged at 4000g for 30min at 4°C. The 25 μL supernatants of each sample was transformed into a new 96-well plate and diluted with 225μL of 50% methanol, 50ul of each mixture was mixed with QC. The 60μL supernatant was transferred to 96-well microplate and sealed with membrane for the LC-MS analysis.

### LC-MS analysis

LC-MS analysis was performed as previously described [50]. All samples were analyzed by the LC-MS system complying the machine orders. The ultra-performance liquid chromatography (UPLC) system was used for chromatographic separations. The reversed phase separation was performed using ACQUITY UPLC BEH C_18_ column (100mm*2.1mm, 1.7μm, Waters, UK) under the conditions of 50°C. The mobile phase solvent A and B were water /0.1% formic acid and acetonitrile / 0.1% formic acid, respectively. Gradient elution was as follows: t = 0–2 min,100% phase A; t = 2–11 min,0% to 100% B; t = 11–13 min,100% B; t = 13–15 min,100% A. The flow rate was 0.4 ml/min. The metabolites eluted form the column were detected by high-resolution tandem mass spectrometer Xevo G2 XS QTOF (Waters, UK). The quadrupole time of flight (Q-TOF) was managed in both positive and negative ion modes. For positive ion mode, the capillary and sampling cone voltages were set to 3,000 V and 40.0 V, respectively, while the negative ion mode was under the condition of 2,000 V and 40.0 V, severally. The mass spectrometry data were gained in Centroid MSE mode. The TOF mass scope was from 50 to 1200 Da under the scan time of 0.2 s. For the MS/MS detection, all precursors were fragmented under following order of 20–40 eV, and the 0.2 s scan time. Moreover, the mass accuracy was performed for LE signal every 3 s during the acquisition. Meanwhile, the quality control samples are collected once every 10 samples to evaluate the stability of the instrument state during sample collection.

### Metabolomics’ data statistics analysis

The predominant aim of metabolomics analysis is to screen statistically and biologically significant metabolites from a large number of detected metabolites. The raw data was performed the KNN method to fill in missing values and removed low-quality ions, QC-RSC (control–based robust LOESS signal correction) was used to correct the data, and delete the corrected data with a relative standard deviation (RSD)> 30% for the statistical analysis. Metabolomics data were initially to detect metabolic feature and chromatographic matching. For LC-MS analysis, each m/z value and MS/MS spectrum of the ionization product were matched in the database including METLIN database (http://metlin.scripps.edu/), HMDB database (http://www.hmdb.ca/), and Lipid Maps database (http:// www.lipidmaps.org/) with parameters of ppm = 10, adducts = [M-H]^-^ and [M+Cl]^-^, and negative ion mode. Metabolomics data needs to be analyzed multi-angle by single-dimensional and multi-dimensional methods, since the data with the feature of high-dimensionality and massive quantity. Differential metabolites between wild type and mutants are analyzed with univariate and multivariate analysis including parametric and nonparametric tests, fold change analysis (FC analysis), principal component analysis (PCA), partial bias Least Squares Discriminant Analysis (PLS-DA). The Variable Importance in Projection (VIP) values of the first two principal components of the multivariate PLS-DA model, combined with fold-change and q-value values of univariate analysis to screen differential metabolites. VIP value exceeding 1.0, fold-change ≥ 1.2 or ≤ 0.8333 and Student’ s t-test P values of less than 0.05 were confirmed to show a significant difference. Statistical tests were constructed via R 3.0.3 software. Differentially accumulated metabolites were analyzed using the KEGG database for pathway analysis. The volcano plot was based on the differential metabolites with two indicators of fold change and q-value. Usually, the fold change is ≥1.2 or ≤0.8333, and q-value is less than 0.05. The relationship among metabolites for constructing metabolic pathways was based on the KEGG database. Cluster analysis of the selected differential metabolites was shown as heat map by R language package (www.r-project.org).

### Transmission election microscopy (TEM)

The epidermis of abdominal fifth segment (the star spots) was dissected from 5th instar larvae 72 ± 3 h after ecdysis. After fat body and muscle attached to epidermis were carefully removed, specimens were fixed immediately with 2.5% glutaraldehyde in phosphate buffer (0.1 M, pH 7.0) for overnight at 4°C, then washed three times with phosphate buffer for 15 min in each time. The processed specimens were further fixed with 1% OsO4 in phosphate buffer for 1–2 h and dehydrated by graded ethanol (30%, 50%, 70%, 80%, 90% and 95%) for about 15 min at each step, then dehydrated by alcohol for 20 min. Afterwards, samples were transferred to absolute acetone for 20 min. After dehydration, the samples were orderly infiltrated in 1:1 mixture of absolute acetone and the final Spurr resin mixture at room temperature for 1 h, followed transferred to 1:3 mixture of absolute acetone and the final resin mixture for 3 h and finally embedded in Spurr resin for overnight at room temperature. Then, the specimens were sectioned with a Leica EM UC7 ultra microtome (Leica, Germany), stained with alkaline lead citrate and uranyl acetate for 5–10 min. Finally, the sections were observed with a transmission electron microscope named Hitachi Model H-7650 (Hitachi, Tokyo, Japan).

### Statistics analysis

Student’s t test and analysis of variance were used to analyze the experimental data. For the Student’s t test, *, p <0.05; **, p < 0.01; ***, p <0.001. Throughout the study, values are represented as the mean ± SE of more than three independent experiments. The statistical analysis of metabolomics data was analyzed using the commercial software Progenesis QI (version 2.2) and the metabolomics R software package meta X.

## Supporting information

S1 Fig Alignment of the amino acid sequences of IDGF among distantly related species. The amino acid sequences of IDGF were compared among the selected species. The lines above the sequences indicate the motifs 1–12.
(TIF)

S2 Fig Alignment of the partial amino acid sequences of IDGF among the six orders. The amino acid sequences around the position from 160 to 190 were compared among the species. The replacement of Glu by Gln was at position 180. Orange box indicate the position 180 and black box represent amino acid sequences of *D*. *melanogaster*.
(TIF)

S3 Fig CRISPR/Cas9-mediated knockout and comparison among WT and mutants. (A) Synoptic depiction of the *BmIDGF* gene structure and small guide RNA (sgRNA) targeting sites. The sgRNA targeting site was located on the sense strand of exon-1. The black rectangle refers to the protein-coding region of BmIDGF, protospacer adjacent motif (PAM) sequences are shown in blue and transcription start sites of T7 promoter are marked by red. (B) The sequence results of homozygote are shown represented via red line, which exhibit 7-bp,8-bp and 9-bp deletion, respectively. The deleted bases are marked by gray letters. (C) Various mutation events around specific targeted sites were confirmed by sequencing. The transcription start site and target site are highlighted in green, the adjacent sequences are in black. Deletions are designated by hyphens. WT: wild type; BmIDGF-7bp, 8bp, 9bp: three kinds of mutant’s line; -indicate deletion.
(TIF)

S4 Fig Expression profiles of *BmIDGF* in different tissues. Relative expression level of *BmIDGF* was determined in the fat body and epidermis by quantitative RT-PCR analysis. *n* = 3. Error bars indicate mean value SEM *p < 0.05, ** p < 0.01, *** p < 0.001 (Student’s t test).
(TIF)

S5 Fig Effect of BmIDGF injection. Expression and purification of the recombinant BmIDGF proteins. His-tagged BmIDGFs were expressed using a baculovirus expression system. The purified BmIDGF proteins were electrophoresed and stained with CBB (A). The purified BmIDGF proteins were analysed by western blotting using the anti-His antibody (B). The *BmIDGF* mutants were injected with PBS (C) or recombinant BmIDGF proteins (D).
(TIF)

S6 Fig The comparison of the uric acid particles and chitinous layer. The enlarged view of same size area from the wild type and mutants, comparing the number of uric acid particles and the thickness of the chitinous layer (A and B). The statistical analysis of the uric acid particles and chitinous layer (C and D). Error bars indicated mean value SEM. Student’s t test, *p < 0.05, ** indicate P-value <0.01. WT, wild type; Mut, mutants.
(TIF)

S7 Fig Expression profiles of melanin synthesis-related genes. The expression patterns of melanin synthesis related genes were determined during the molting stage (A and C, Crescent; B and D, Star spots). The molting period were determined based on the head capsule slippage (HCS) index. AHCS, after head capsule slippage; WT, wild type; Mut, mutants. *n* = 3. Error bars indicate mean value SEM, *p < 0.05, ** p < 0.01, *** p < 0.001 (Student’s t test).
(TIF)

S8 Fig The metabolic profiling reveals distinct changes in metabolism of the *BmIDGF* mutants. PCA score scatter plot depicts metabolic profiles of wild type and mutants at three time points (A). PCA reveals metabolic shifts and each dot represents the metabolic status of six biological replicates. Plots with different colors indicate samples of different time points. WT, wild type; Mut, mutants. Comparison of metabolite abundances between the *BmIDGF* mutants and wild type from fourth instar (B), fourth instar moulting (C) and fifth instar (D). Black and gray circles indicate metabolites that changed significantly (FC >1.2 or <0.833, p-value < 0.05). Red dots are crucial metabolites of the tyrosine metabolism that changed significantly.
(TIF)

S9 Fig Relative levels of the total differential metabolites. Heatmap of all the significant differentially accumulating metabolites between the *BmIDGF* mutant and wild type at three time points. Shades from green to red represent the increasing metabolite levels. 4th, fourth instar; 4m, fourth instar moulting; 5m, fifth instar; WT, wild type; Mut, mutants.
(TIF)

S10 Fig Metabolomic pathway changes following interruption of *BmIDGF*. Schematic map of pathways with significant enrichment score between wild type and mutants at fourth instar (A), fourth instar moulting (B) and fifth instar (C). Enrich score for each metabolic pathway was used to represent the enrichment significance of differential metabolites in this pathway. An analysis of variance was used for the statistical test (P < 0.05 indicates significance). Different color circles represent the -log10(p-value) values of different metabolic pathways. 4th, fourth instar; 4m, fourth instar moulting; 5m, fifth instar.
(TIF)

S1 Table List of species, gene name and accession number. (DOCX)

S2 Table List of primers used in this paper. (DOCX)
