# Supplementary figures and images for: Imaginal disc growth factor maintains cuticle structure and controls melanization in the spot pattern formation of Bombyx mori
Source: PLoS Genet. 2020 Sep 28;16(9):e1008980. doi: 10.1371/journal.pgen.1008980 (PMC7544146; doi:10.1371/journal.pgen.1008980)

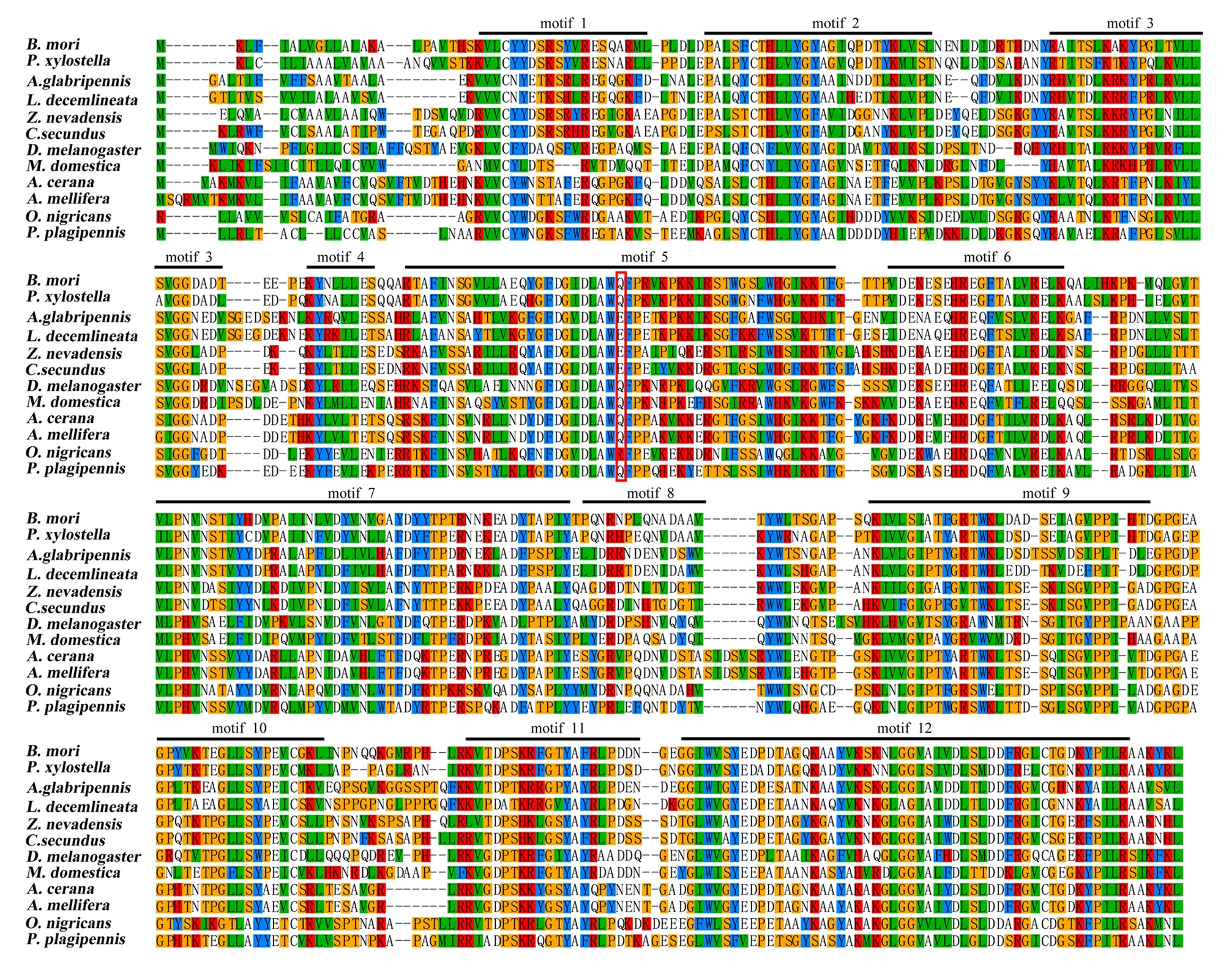

Supplement: S1 Fig — The amino acid sequences of IDGF were compared among the selected species. The lines above the sequences indicate the motifs 1–12. (TIF) [file pgen.1008980.s001.tif]

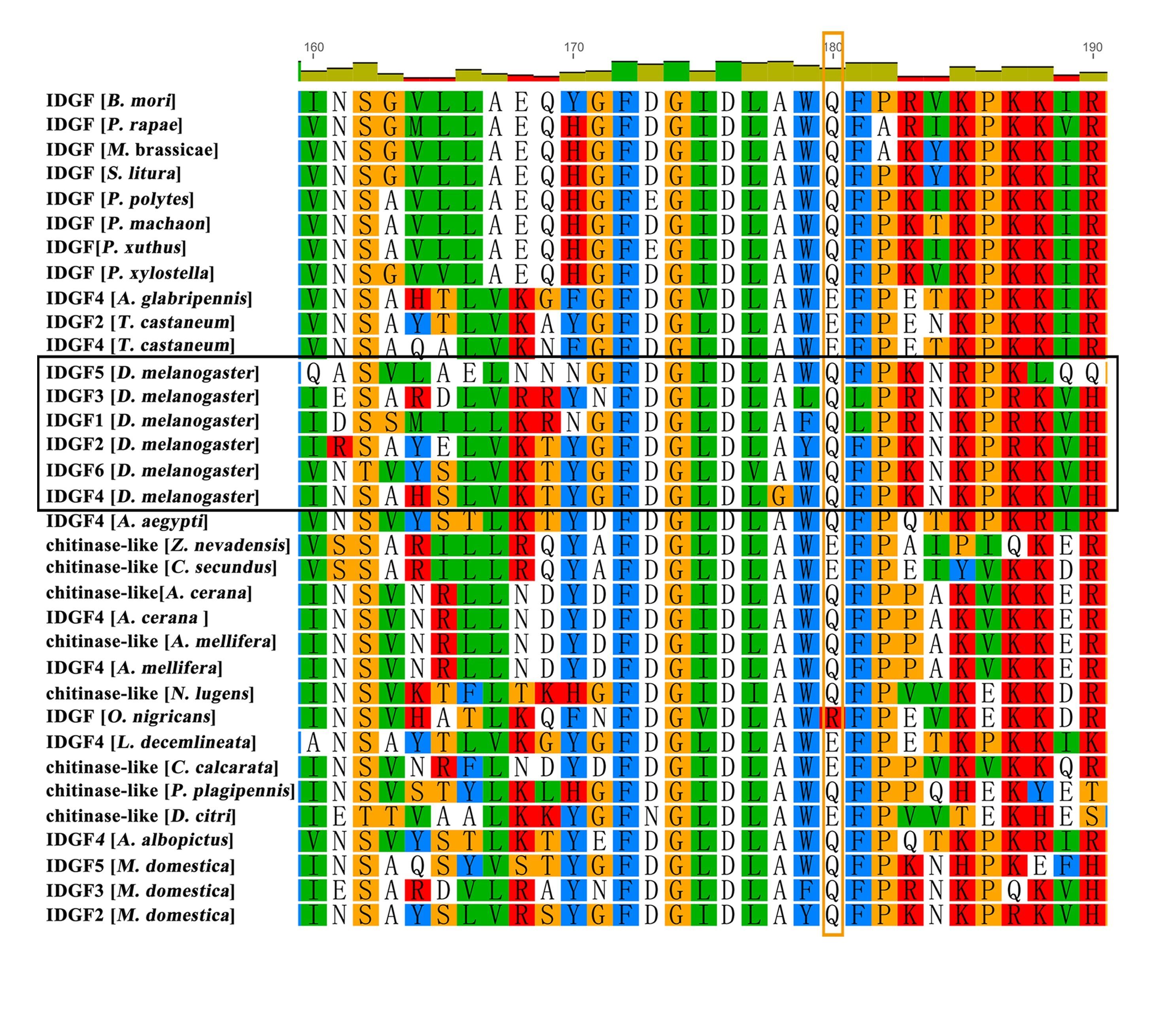

Supplement: S2 Fig — The amino acid sequences around the position from 160 to 190 were compared among the species. The replacement of Glu by Gln was at position 180. Orange box indicate the position 180 and black box represent amino acid sequences of D. melanogaster. (TIF) [file pgen.1008980.s002.tif]

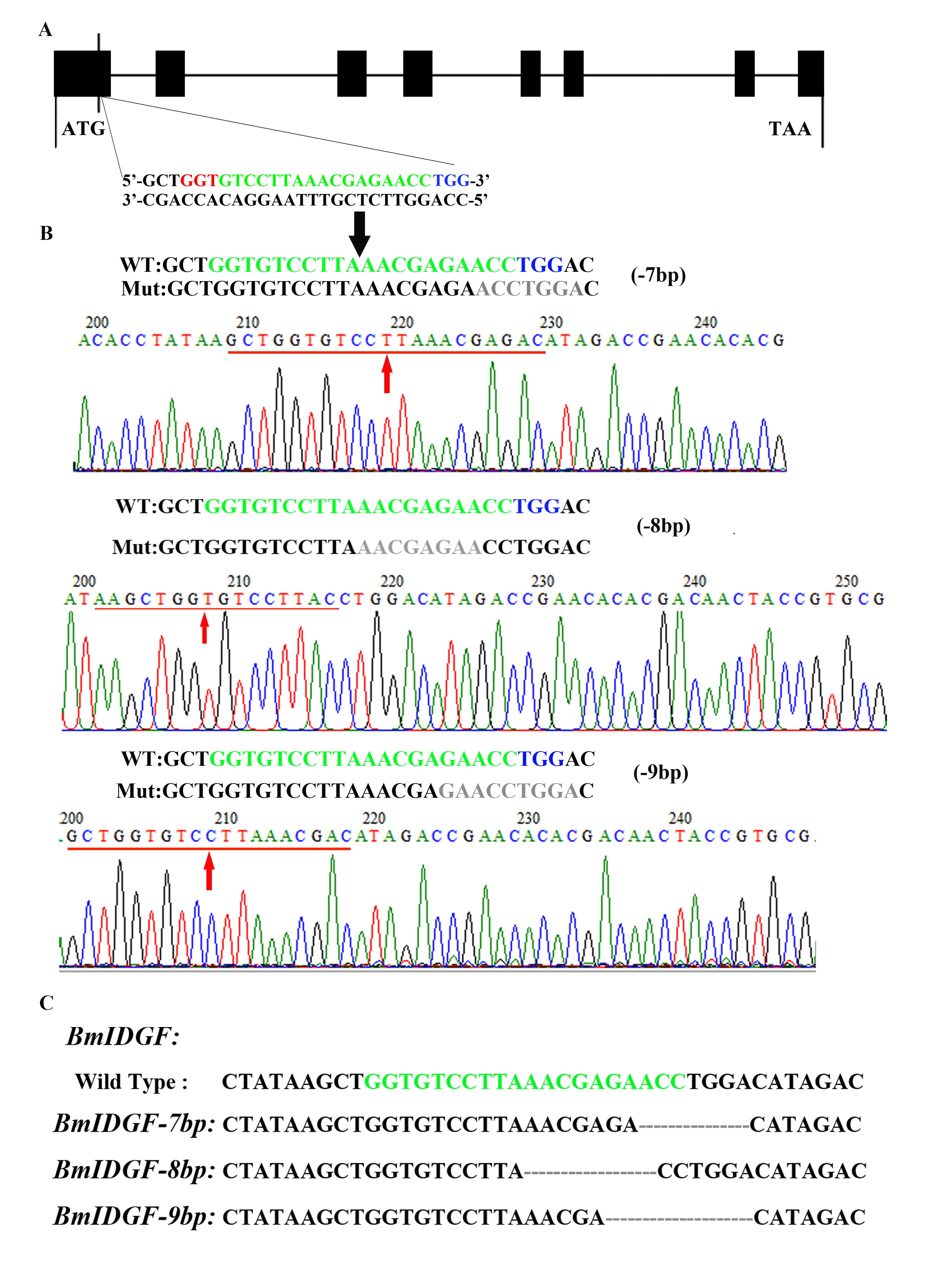

Supplement: S3 Fig — (A) Synoptic depiction of the BmIDGF gene structure and small guide RNA (sgRNA) targeting sites. The sgRNA targeting site was located on the sense strand of exon-1. The black rectangle refers to the protein-coding region of BmIDGF, protospacer adjacent motif (PAM) sequences are shown in blue and transcription start sites of T7 promoter are marked by red. (B) The sequence results of homozygote are shown represented via red line, which exhibit 7-bp,8-bp and 9-bp deletion, respectively. The deleted bases are marked by gray letters. (C) Various mutation events around specific targeted sites were confirmed by sequencing. The transcription start site and target site are highlighted in green, the adjacent sequences are in black. Deletions are designated by hyphens. WT: wild type; BmIDGF-7bp, 8bp, 9bp: three kinds of mutant’s line; -indicate deletion. (TIF) [file pgen.1008980.s003.tif]

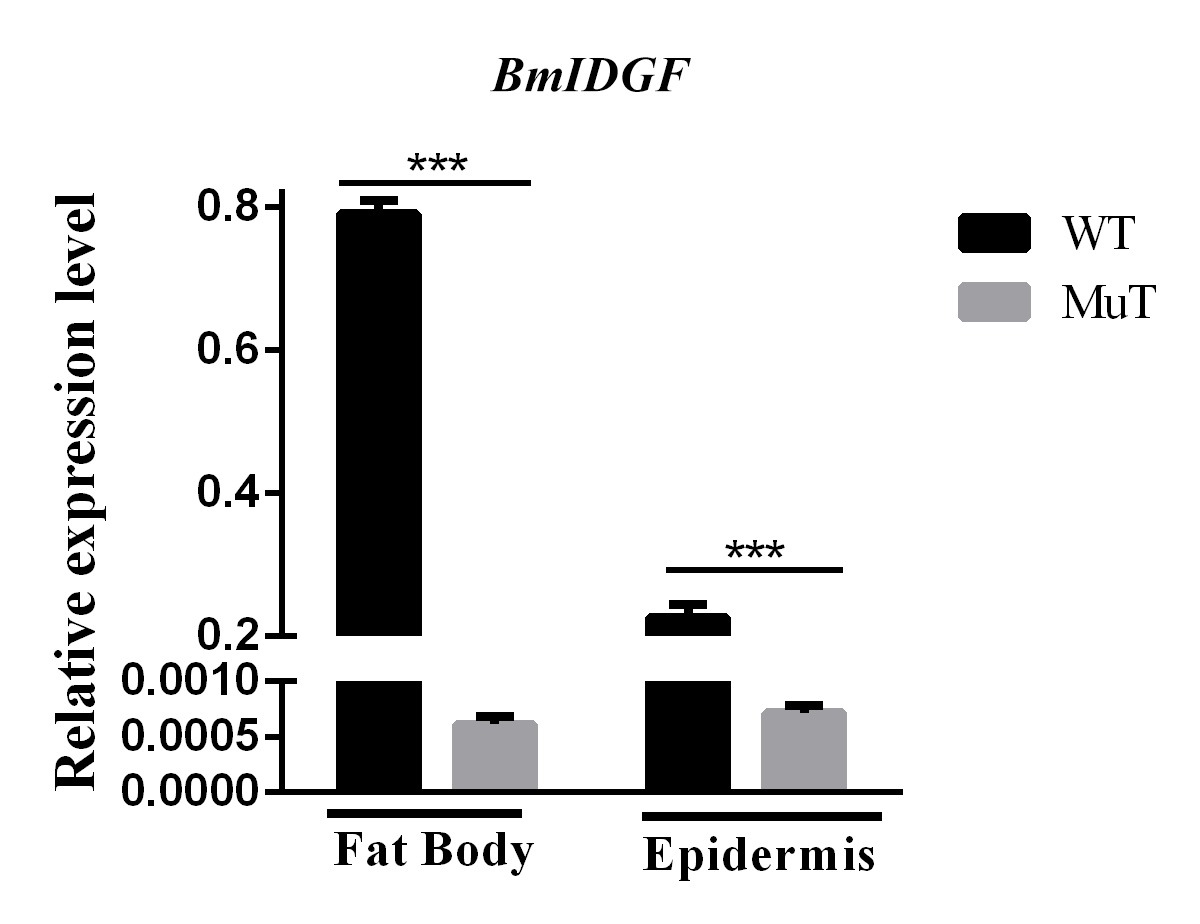

Supplement: S4 Fig — Relative expression level of BmIDGF was determined in the fat body and epidermis by quantitative RT-PCR analysis. n = 3. Error bars indicate mean value SEM *p < 0.05, ** p < 0.01, *** p < 0.001 (Student’s t test). (TIF) [file pgen.1008980.s004.tif]

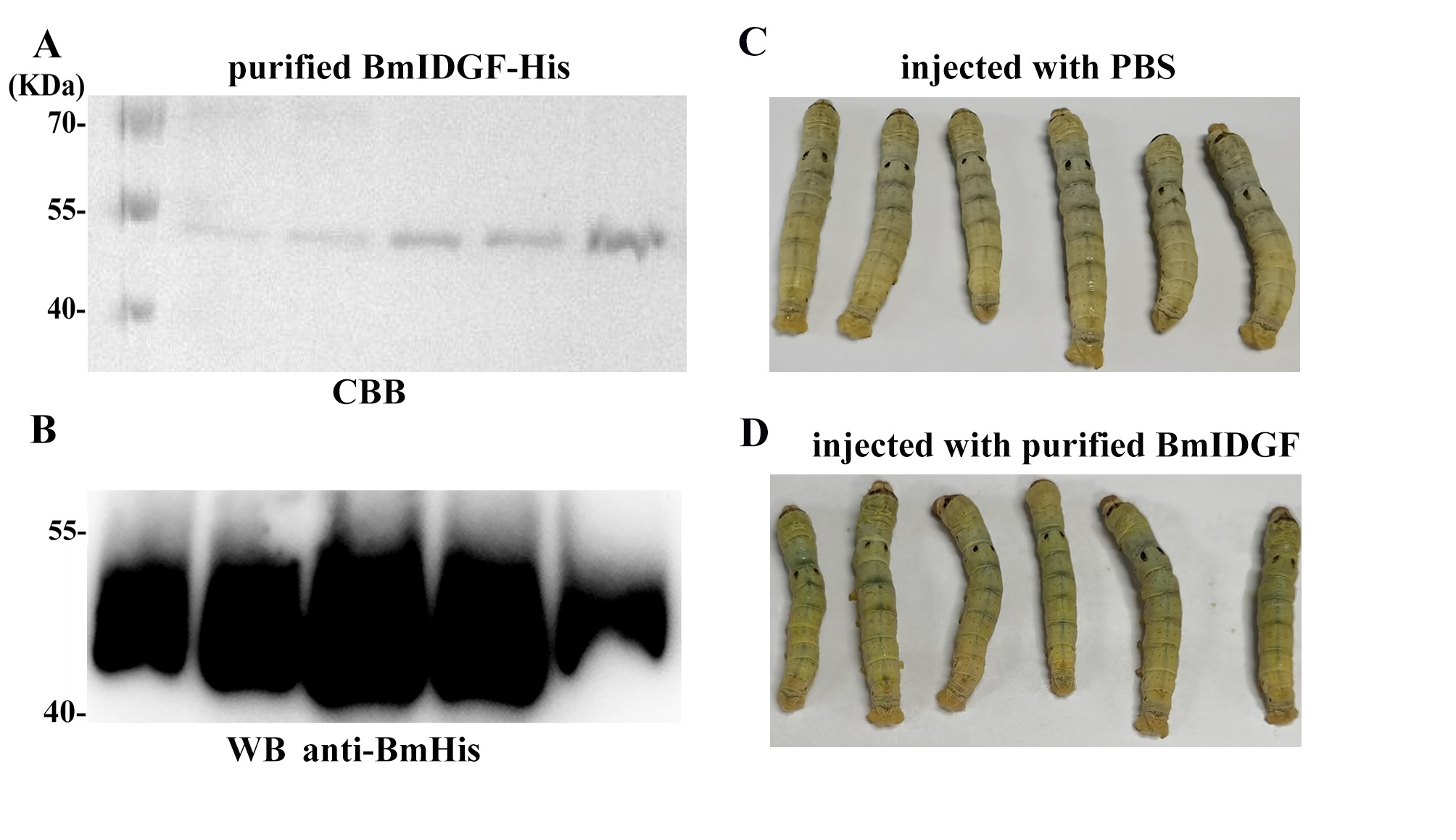

Supplement: S5 Fig — Expression and purification of the recombinant BmIDGF proteins. His-tagged BmIDGFs were expressed using a baculovirus expression system. The purified BmIDGF proteins were electrophoresed and stained with CBB (A). The purified BmIDGF proteins were analysed by western blotting using the anti-His antibody (B). The BmIDGF mutants were injected with PBS (C) or recombinant BmIDGF proteins (D). (TIF) [file pgen.1008980.s005.tif]

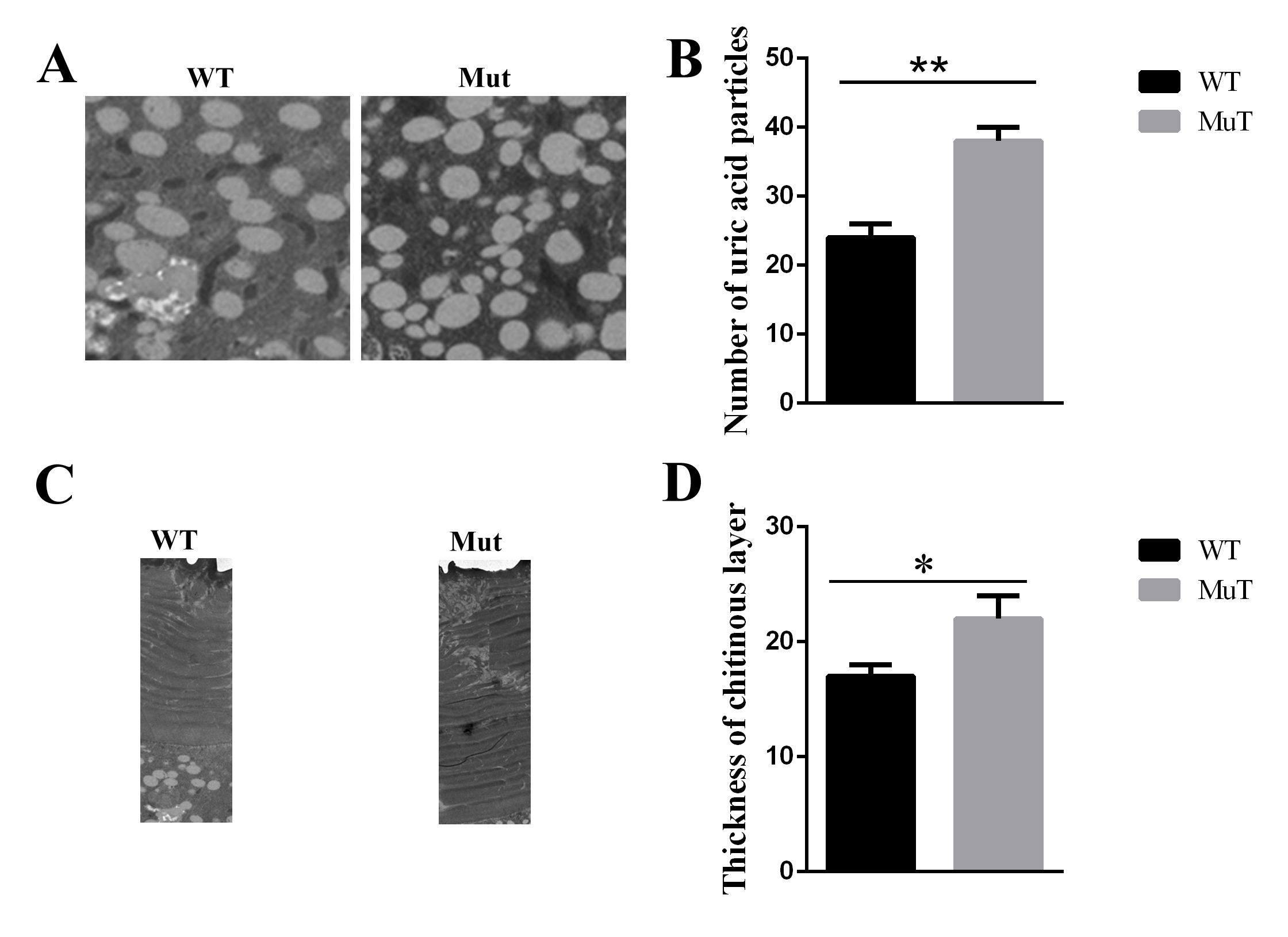

Supplement: S6 Fig — The enlarged view of same size area from the wild type and mutants, comparing the number of uric acid particles and the thickness of the chitinous layer (A and B). The statistical analysis of the uric acid particles and chitinous layer (C and D). Error bars indicated mean value SEM. Student’s t test, *p < 0.05, ** indicate P-value <0.01. WT, wild type; Mut, mutants. (TIF) [file pgen.1008980.s006.tif]

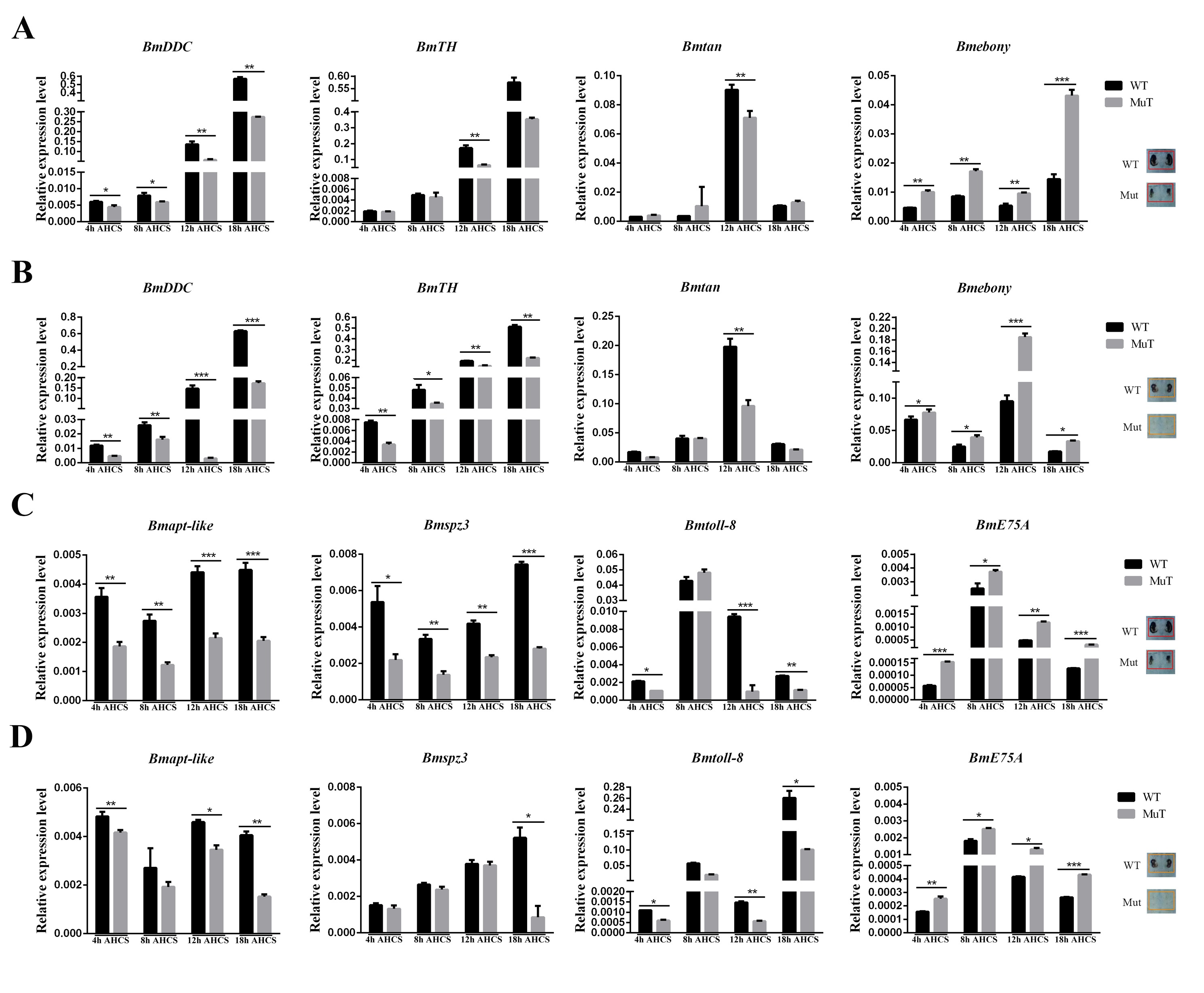

Supplement: S7 Fig — The expression patterns of melanin synthesis related genes were determined during the molting stage (A and C, Crescent; B and D, Star spots). The molting period were determined based on the head capsule slippage (HCS) index. AHCS, after head capsule slippage; WT, wild type; Mut, mutants. n = 3. Error bars indicate mean value SEM, *p < 0.05, ** p < 0.01, *** p < 0.001 (Student’s t test). (TIF) [file pgen.1008980.s007.tif]

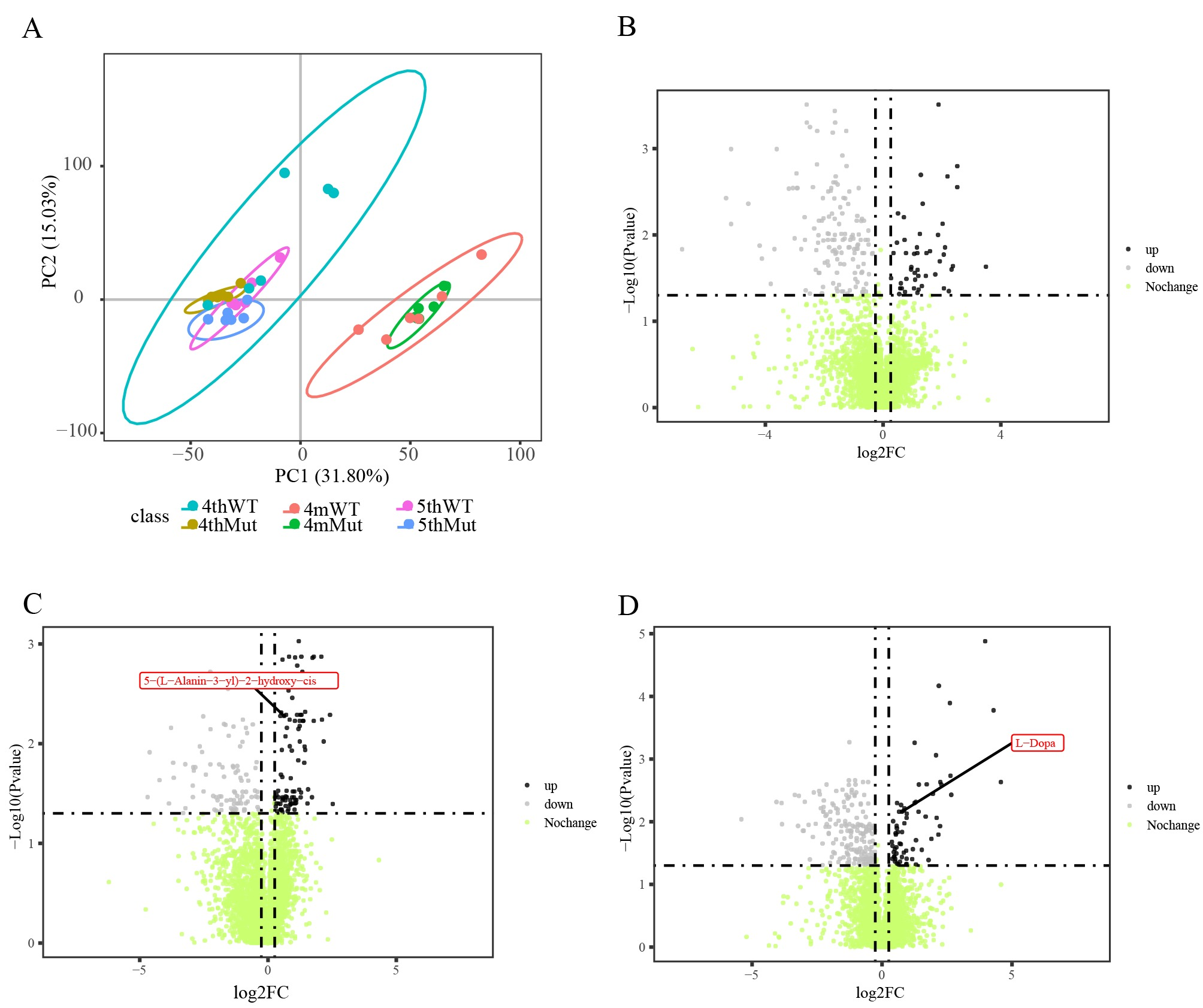

Supplement: S8 Fig — PCA score scatter plot depicts metabolic profiles of wild type and mutants at three time points (A). PCA reveals metabolic shifts and each dot represents the metabolic status of six biological replicates. Plots with different colors indicate samples of different time points. WT, wild type; Mut, mutants. Comparison of metabolite abundances between the BmIDGF mutants and wild type from fourth instar (B), fourth instar moulting (C) and fifth instar (D). Black and gray circles indicate metabolites that changed significantly (FC >1.2 or <0.833, p-value < 0.05). Red dots are crucial metabolites of the tyrosine metabolism that changed significantly. (TIF) [file pgen.1008980.s008.tif]

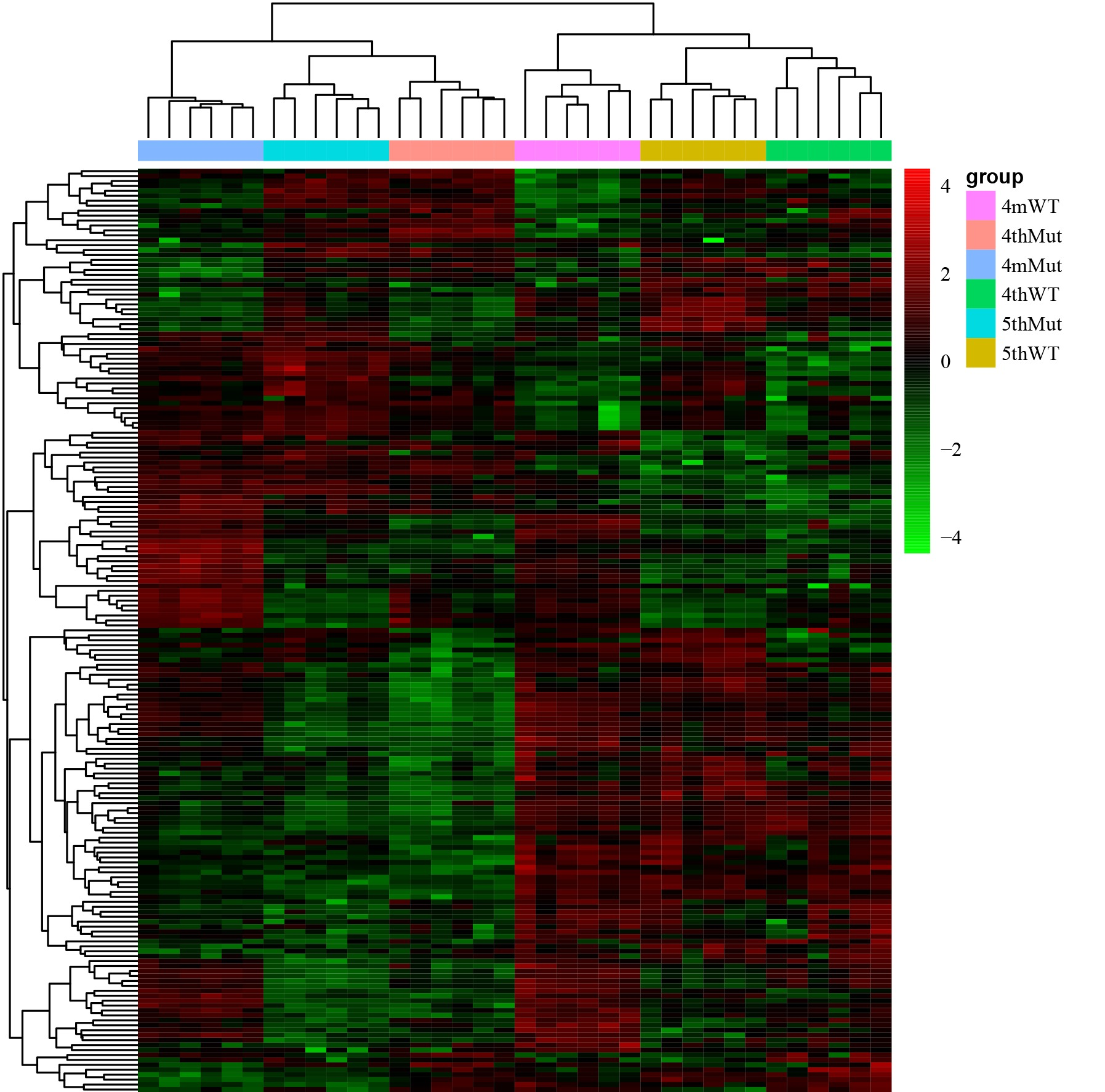

Supplement: S9 Fig — Heatmap of all the significant differentially accumulating metabolites between the BmIDGF mutant and wild type at three time points. Shades from green to red represent the increasing metabolite levels. 4th, fourth instar; 4m, fourth instar moulting; 5m, fifth instar; WT, wild type; Mut, mutants. (TIF) [file pgen.1008980.s009.tif]

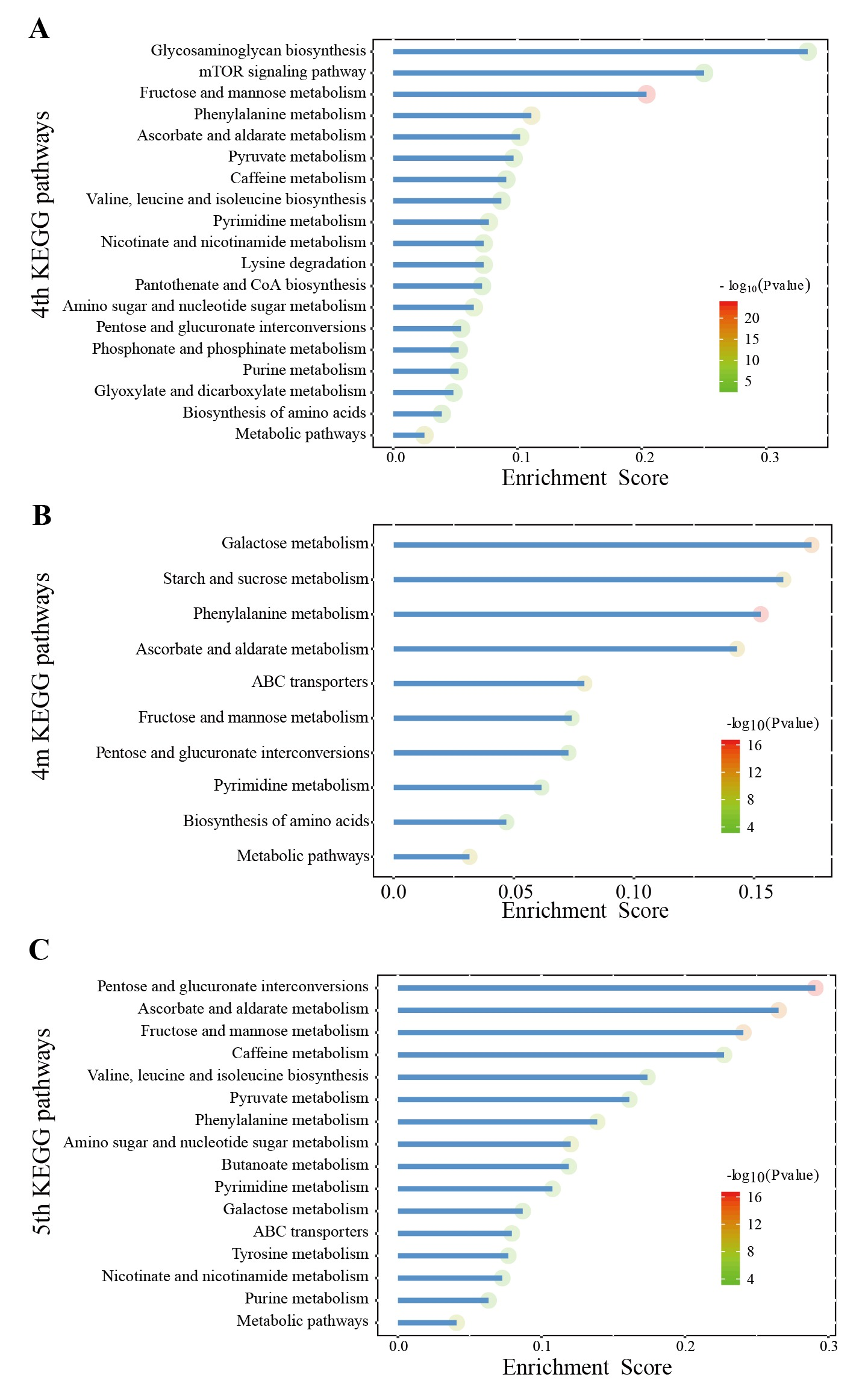

Supplement: S10 Fig — Schematic map of pathways with significant enrichment score between wild type and mutants at fourth instar (A), fourth instar moulting (B) and fifth instar (C). Enrich score for each metabolic pathway was used to represent the enrichment significance of differential metabolites in this pathway. An analysis of variance was used for the statistical test (P < 0.05 indicates significance). Different color circles represent the -log10(p-value) values of different metabolic pathways. 4th, fourth instar; 4m, fourth instar moulting; 5m, fifth instar. (TIF) [file pgen.1008980.s010.tif]
