# Supplementary material for: Imaginal disc growth factor maintains cuticle structure and controls melanization in the spot pattern formation of Bombyx mori
Source: PLoS Genet. 2020 Sep 28;16(9):e1008980. doi: 10.1371/journal.pgen.1008980 (PMC7544146; doi:10.1371/journal.pgen.1008980)
Supplement: S1 Table — (DOCX) [file pgen.1008980.s011.docx]

**S1 Table. List of species, gene name and accession number**

| **Species** | **Gene name** | **Accession number** |
| --- | --- | --- |
| *Bombyx mori* | *BmorIDGF* | AB183872.1 |
| *Mamestra brassicae* | *MbraDGF* | DQ355162.1 |
| *Spodoptera litura* | *Slitchitinase-like protein EN03* | XM_022978565.1 |
| *Papilio polytes* | *Ppolchitinase-like protein EN03* | NM_001311400.1 |
| *Papilio machaon* | *Pmacchitinase-like protein EN03* | XM_014510987.1 |
| *Papilio Xuthus* | *Pxutchitinase-like protein EN03* | NM_001312778.1 |
| *Plutella xylostella* | *PxylIDGF* | AB282642.1 |
| *Anoplophora glabripennis* | *AglaIDGF4* | XM_018715827.1 |
| *Tribolium castaneum* | *TcasIDGF2* | NM_001044627.1 |
| *Tribolium castaneum* | *TcasIDGF4* | NM_001044626.1 |
| *Drosophila melanogaster* | *DmelIDGF1* | AF394708.1 |
| *Drosophila melanogaster* | *DmelIDGF2* | AF102237.1 |
| *Drosophila melanogaster* | *DmelIDGF3* | AF394718.1 |
| *Drosophila melanogaster* | *DmelIDGF4* | NM_001298139.1 |
| *Drosophila melanogaster* | *DmelIDGF5* | NM_137477.4 |
| *Drosophila melanogaster* | *DmelIDGF6* | NM_057733.4 |
| *Aedes aegypti* | *AaegIDGF4* | XM_001660695.2 |
| *Zootermopsis nevadensis* | *Znevchitinase-like protein* | XM_022084000.1 |
| *Cryptotermes secundus* | *Csecchitinase-like protein* | XM_023870763.2 |
| *Apis cerana* | *AcerIDGF4* | XM_017054438.2 |
| *Apis cerana* | *Acer chitinase-like protein* | XM_017054439.2 |
| *Apis mellifera* | *Amelchitinase-like protein* | XM_016913525.1 |
| *Apis mellifera* | *AmelIDGF4* | XM_016913527.2 |
| *Nilaparvata lugens* | *Nlugchitinase-like protein EN03* | XM_022339149.1 |
| *Oncometopia nigricans* | *OnigIDGF* | AY725777.1 |
| *Leptinotarsa decemlineata* | *LdecIDGF4* | XM_023158368.1 |
| *Ceratina calcarata* | *Ccalchitinase-like protein EN03* | XM_018020691.2 |
| *Pristhesancus plagipennis* | *Pplachitinase-like protein EN03* | KY030994.1 |
| *Diaphorina citri* | *Dcitchitinase-like protein EN03* | XM_017449611.1 |
| *Aedes albopictus* | *AalbIDGF4* | XM_020075376.2 |
| *Musca domestica* | *MdomIDGF2* | XM_005179351.3 |
| *Musca domestica* | *MdomIDGF3* | XM_005179352.3 |
| *Musca domestica* | *MdomIDGF5* | XM_005186242.3 |
| *Frankliniella occidentalis* | *FoccIDGF4* | XM_026420798.1 |
| *Frankliniella occidentalis* | *Foccchitinase-like protein* | XM_026436107.1 |
| *Hydropsyche angustipennis* | *HangIDGF* | MN520322.1 |
| *Plectrocnemia conspersa* | *PconIDGF1* | MN520319.1 |
| *Plectrocnemia conspersa* | *PconIDGF2* | MN520320.1 |
| *Plectrocnemia conspersa* | *PconIDGF3* | MN520321.1 |
| *Glyphotaelius pellucidus* | *GpelIDGF* | MN520337.1 |
| *Oligotricha striata* | *OstrIDGF* | MN520323.1 |
| *Rhyacophila obliterata* | *RoblIDGF* | MN520324.1 |
| *Bombyx mori* | *BmorCHI* | NM_001044015.1 |
| *Bombyx mori* | *BmorCHI-h* | NM_001114935.1 |
| *Spodoptera litura* | *SlitCHI* | AY325497.1 |
| *Mamestra brassicae* | *MbraCHI* | JN558350.1 |
| *Papilio polytes* | *PpolCHI* | NM_001311726.1 |
| *Plutella xylostella* | *PxylCHI* | MH899223.1 |
