# Supplementary material for: Imaginal disc growth factor maintains cuticle structure and controls melanization in the spot pattern formation of Bombyx mori
Source: PLoS Genet. 2020 Sep 28;16(9):e1008980. doi: 10.1371/journal.pgen.1008980 (PMC7544146; doi:10.1371/journal.pgen.1008980)
Supplement: S2 Table — (DOCX) [file pgen.1008980.s012.docx]

**S2 Table. List of primers used in this paper**

| **Primer name** | **Sequence (5’-3’)** | **Purpose** |
| --- | --- | --- |
| BmIDGFCRI  SPR/Cas9-F | GAAATTAATACGACTCACTATA  GTGGTGTCCTTAAACGAGAA  CCTTTAGAGCTAGAAATAGC | For the synthesis of sgRNA template |
| BmIDGFCRI  SPR/Cas9-R | AAAAGCACCGACTCGGTGCCACTTTTT  CAAGTTGATAACGGACTAGCCTTATT  TTAACTTGCTATTTCTAGCTCTAAAAC | For the synthesis of sgRNA template |
| BmIDGFcas9-cheF | GCTTTTACTCGCTTATCTCCAG | detect mutants |
| BmIDGFcas9-cheR | TATCAATCGCATTCACTTACCA | detect mutants |
| BmIDGF-QF | ACAAAATCGCAACCCACTGC | qRT-PCR of *BmIDGF* |
| BmIDGF-QR | AGATACGACCGGAGCCGA | qRT-PCR of *BmIDGF* |
| BmIDGF-ORF-F | CGGAATTCATGAAGCTATTTATCGCTCT | cloning of BmIDGF into pFastbac |
| BmIDGF-ORF-R | CCGCTCGAGTTAATGGTGATGGTGATGATGGAGACGGTATTTAGCGGCC | cloning of BmIDGF into pFastbac |
| BmTH-QF | CGCCTTTCCACACACCTGAACC | qRT-PCR of *BmTH* |
| BmTH-QR | GGGATGCAAGGCCAATTTCTTGCG | qRT-PCR of *BmTH* |
| BmDDC-QF | GATGAAGACATCCGCAACGGTCTCA | qRT-PCR of *BmDDC* |
| BmDDC-QR | ATCTCCAATTTCGTCAAGAGCGTCG | qRT-PCR of *BmDDC* |
| Bmtan-QF | TCAAACAAACCAAGCAGAGC | qRT-PCR of *Bmtan* |
| Bmtan-QR | TCGCCTATATCAGCATCAGC | qRT-PCR of *Bmtan* |
| Bmebony-QF | CGTTTATACCGCACAGGAGA | qRT-PCR of *Bmebony* |
| Bmebony-QR | GTAGCATAGGACCACGCACT | qRT-PCR of *Bmebony* |
| Bmspz3-QF | CAAAACACAGCTCCACGCGGTAC | qRT-PCR of *Bmspz3* |
| Bmspz3-QR | CCGTTTCGTAGAAGTATCGGACCTTG | qRT-PCR of *Bmspz3* |
| BmToll8-QF | CAAGCATTGTCGGTTCTTTCTG | qRT-PCR of *BmToll8* |
| BmToll-8-QR | TTTCGGAGTGCTTGTGGATTT | qRT-PCR of *BmToll8* |
| BmE75A-QF | GCTTGTCGGTCGGGATGAGTC | qRT-PCR of *BmE75A* |
| BmE75A-QR | TTCTCCGCAGTTGTTCGTGTT | qRT-PCR of *BmE75A* |
| Bmapt-like-QF | TAGCCAGACATAGGGACGTACTCC | qRT-PCR of *Bmapt-like* |
| Bmapt-like-QR | TTGACTTGCGCGGGGGTC | qRT-PCR of *Bmapt-like* |
| Bmrpl49-F | CAGGCGGTTCAAGGGTCAATAC | qRT-PCR of *Bmrpl49* |
| Bmrpl49-R | TGCTGGGCTCTTTCCACGA | qRT-PCR of *Bmrpl49* |
